# Health-related quality of life in immunocompromised adults with mild–moderate COVID-19 treated with nirmatrelvir-ritonavir: results from the randomized, double-blinded EPIC-IC trial

**DOI:** 10.1186/s12955-026-02518-8

**Published:** 2026-03-19

**Authors:** Ruth Mokgokong, Paul Cislo, Elena Tudone, Edward Weinstein, Joseph C. Cappelleri

**Affiliations:** 1 Pfizer Ltd, Walton Oaks, Tadworth, KT20 7NS UK; 2 Pfizer Inc, New York, NY USA; 3 Pfizer s.r.l., Milan, Italy; 4 Pfizer Inc, Groton, Connecticut USA

**Keywords:** Antiviral agents, COVID-19, COVID-19 drug therapy, EPIC-IC, Health-related quality of life, Immunocompromised patient, Nirmatrelvir/ritonavir, Patient-reported outcomes, SARS-CoV-2, Viral protease inhibitors

## Abstract

**Background:**

Little is known about health-related quality of life (HRQoL) in immunocompromised people during and after COVID-19 illness. We describe HRQoL outcomes from the EPIC-IC trial, which included participants with immunocompromise and mild–moderate COVID-19.

**Methods:**

EPIC-IC was a randomized, double-blind trial. Participants were assigned 1:1:1 to 5-day, 10-day, or 15-day nirmatrelvir-ritonavir (NMV/r) and completed the SF-36 and EQ-5D-5L through Week (W)24. HRQoL was analyzed across and by treatment arms for the evaluable population (*N* = 150) and in post hoc subpopulations with severe (*n* = 57) and non-severe (*n* = 93) immunocompromise. Mixed-effects longitudinal models compared 5-day vs. 10-day or 15-day NMV/r.

**Results:**

In the overall sample, mean baseline SF-36 domain scores (norm-based; mean = 50, SD = 10) ranged from 35.7 to 44.1, mean Physical Component Summary (PCS) score was 37.9, and mean Mental Component Summary (MCS) score was 41.5 – all substantially worse than cancer comparator norms and age-matched general population (AMGP) norms. Baseline mean EQ-5D-5L Index score was 0.65; participants reported problems with pain/discomfort (90% of participants), usual activities (73%), mobility (50%), anxiety/depression (48%), and self-care (29%). These proportions improved through Day (D)15 (change from baseline [CFB]: − 49%-points for pain/discomfort, − 38%-points usual activities, − 22%-points mobility, − 24%-points anxiety/depression, − 19%-points self-care) and then stabilized. From the earliest post-baseline assessment (EQ-5D-5L: D5, SF-36: D10), all overall-sample outcomes improved significantly (-5D-5*p* < 0.05), with all mean improvements exceeding published minimum important difference thresholds (SF-36: 2–4-point; EQ-5D-5L Index: 0.03–0.05-point). Mean EQ-5D-5L Index score surpassed AMGP norms from D5 and peaked at D15 (0.21-point CFB). Mean SF-36 outcomes improved through W12; PCS surpassed AMGP norms at W12, while MCS approached but remained below AMGP norms. Improvements were sustained through W24 (W24 CFB: PCS, 10-point; MCS, 8-point; EQ-5D-5L Index, 0.20-point). EQ-5D-5L improvements appeared similar across immunocompromised subpopulations, whereas SF-36 improvements appeared slower and smaller in severely vs. non-severely immunocompromised participants. No significant treatment-arm differences were observed, except lower D10 PCS scores among severely immunocompromised participants treated with 5-day vs. 10-day NMV/r (*p* = 0.03).

**Conclusion:**

Immunocompromised individuals with mild–moderate COVID-19 experienced various HRQoL decrements, followed by rapid improvements during treatment that were sustained through W24. Interpretation is limited by the absence of an untreated control group.

**Clinical trial registration:**

Clinical trial number: NCT05438602. Registry: ClinicalTrials.gov. Registration date: June 28, 2022. URL: https://clinicaltrials.gov/ct2/show/NCT05438602.

**Supplementary Information:**

The online version contains supplementary material available at 10.1186/s12955-026-02518-8.

## Plain language summary

People with weakened immune systems (called immunocompromised) are at higher risk of serious illness from COVID-19, but little is known about how they feel and function during COVID-19 illness or recovery. This study looked at how COVID-19 affected the health and well-being of immunocompromised people who took part in the EPIC-IC clinical trial. In this trial, immunocompromised participants with mild to moderate COVID-19 were randomly chosen to receive 5, 10, or 15 days of the antiviral medicine nirmatrelvir-ritonavir (NMV/r). Participants completed questionnaires about their health-related quality of life (HRQoL) when they joined the study and during 6 months of follow-up. A questionnaire called the EQ-5D-5L asked participants 5 questions about their current pain or discomfort, anxiety or depression, and how well they could care for themselves, get around, and do their usual activities. Another questionnaire, called the SF-36, asked participants 36 questions about their physical and mental health over the last week and how their health has affected their daily and social activities. At the start of the study (during the first days of COVID-19 illness), participants reported much poorer health and functioning than people of similar age in the general population and people with cancer. Common problems included pain, fatigue, difficulty doing usual activities, and anxiety/depression. On average, participants showed clinically meaningful improvements across all parts of HRQoL within the first 2 weeks of treatment, and these gains were maintained through 6 months. Physical health scores reached population averages after 12 weeks in the study, whereas mental health scores improved but stayed somewhat below average. People with severe immune problems had better physical health scores at Day 10 of the study if they had been chosen to take 10 days instead of 5 days of treatment. Overall, people with immune problems had quick and long-lasting recovery of HRQoL during and after NMV/r treatment. The length of NMV/r treatment (5, 10, or 15 days) did not make a major difference in HRQoL recovery.

**Supplementary Information:**

The online version contains supplementary material available at 10.1186/s12955-026-02518-8.

## Background

COVID-19 illness can substantially impact health-related quality of life (HRQoL) in the general population [1–8], even when COVID-19 severity is mild [3, 8] or mild–moderate [7, 8]. Immunocompromised individuals are at greater risk for prolonged SARS-CoV-2 infection and severe clinical outcomes from COVID-19 than individuals without immunocompromise (IC) [9–13]. Several treatments are available to reduce the risk of severe outcomes in immunocompromised populations, including the antiviral nirmatrelvir-ritonavir (NMV/r) [14, 15]. However, very few studies have examined how COVID-19 illness impacts HRQoL in people with IC [16, 17]. More evidence is needed on how people with IC perceive their HRQoL during acute COVID-19 and how HRQoL changes over time in this population following COVID-19.

Longitudinal HRQoL data from the Phase 2 EPIC-IC trial (NCT05438602) [18] provide a unique opportunity to address these evidence gaps. EPIC-IC enrolled a rigorously defined and clinically verified population with IC and acute mild–moderate COVID-19 and prospectively assessed HRQoL from the first days of infection [18], allowing novel insights into how immunocompromised individuals experience this period of illness. Moreover, participants in EPIC-IC were treated with NMV/r [18], a first-line, guideline-recommended antiviral therapy for high-risk patients with mild–moderate COVID-19 [19–21]; these data therefore provide valuable information on how HRQoL changes during and following antiviral treatment.

EPIC-IC was conducted to assess whether extended NMV/r regimens may provide more benefit than the approved 5-day regimen in immunocompromised individuals [18], given that people with IC may have continued viral and clinical progression of COVID-19 after completing the approved 5-day regimen [18, 22]. Support for the potential benefit of extended regimens comes from a small retrospective study of people with severe IC, in which antiviral therapy of > 10 days was more effective in reducing symptoms and viral load than antiviral therapy of ≤ 5 days [23]. Building on the existing evidence, EPIC-IC randomized immunocompromised participants with acute mild–moderate COVID-19 to treatment with a 5-day, 10-day, or 15-day regimen of NMV/r (1:1:1) and followed viral, clinical, and HRQoL outcomes for 24 weeks [18]. Post hoc analyses explored the benefits of 5-day vs. extended NMV/r in subpopulations with severe IC and non-severe IC [18]. Analyses of sustained viral clearance, the primary endpoint in EPIC-IC, supported similar NMV/r efficacy across the 3 treatment durations [18]. However, viral rebound was nominally more common with 5-day vs. extended NMV/r [18]. Through Day 44, no deaths from any cause were observed, and serious adverse events were reported in 2–9% of participants across arms, with no increase in serious adverse events with longer NMV/r duration [18].

Here, we present novel patient-reported HRQoL data from EPIC-IC [18], which captured HRQoL in immunocompromised individuals at baseline (i.e., during the first days of COVID-19 symptoms) and at several post-baseline visits with the 36-Item Short Form Health Survey version 2 – acute form (SF-36) and the EQ-5D-5L, two widely used, multidimensional measures of HRQoL [24–33]. Our primary aims are to describe HRQoL in immunocompromised adults with mild–moderate COVID-19 and to compare HRQoL before, during, and following treatment with NMV/r. We also aim to compare HRQoL changes between people receiving 5-day vs. extended NMV/r and explore HRQoL changes in subpopulations with severe and non-severe IC.

## Methods

A summary of the EPIC-IC trial methods and results is provided below; additional methods and details of the EPIC-IC trial have been published [17, 18].

### EPIC-IC trial design

**Fig. 1:**
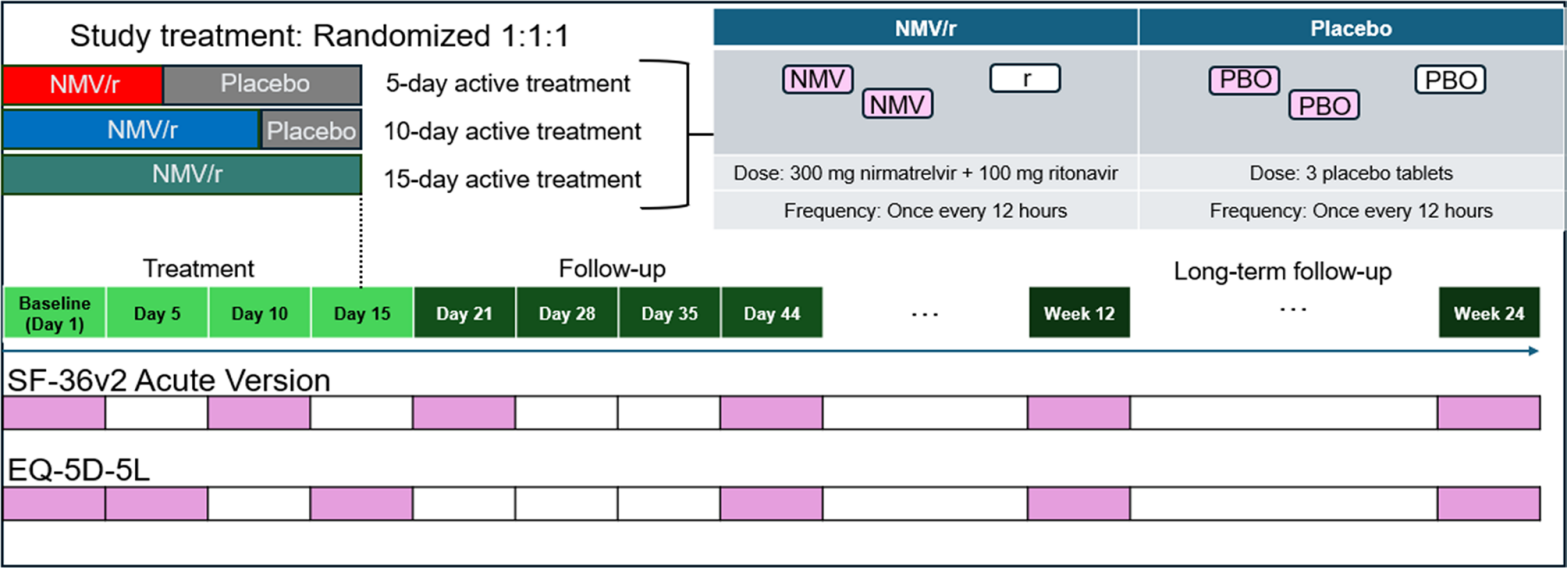
Schedule of treatment and patient-reported outcome assessments in the EPIC-IC trial. Participants were randomized 1:1:1 to receive active treatment with NMV/r for the first 5, 10, or 15 days of the study. Patient-reported outcome assessments were completed on the baseline and post-baseline visit days indicated by pink-shaded bars and could be entered into the electronic study diary on the target day or up to 1 day later. Abbreviations: NMV, nirmatrelvir; NMV/r, nirmatrelvir-ritonavir; PBO, placebo; r, ritonavir; SF-36, 36-Item Short Form Survey version 2

Briefly, immunocompromised participants were randomized 1:1:1 to 5-day, 10-day, or 15-day NMV/r treatment (Fig. 1) [18] to compare the efficacy of the approved 5-day regimen to that of extended regimens. All participants completed a 15-day treatment period on Study Days 1–15; those in the 5-day and 10-day NMV/r arms received placebo after their NMV/r course was completed (Fig. 1). Study visits continued through Week 24 (Fig. 1). Participants and investigators were blinded to participants’ assigned treatment durations throughout the trial [17, 18].

Ethics approvals for the EPIC-IC protocol, amendments, and other relevant documents were granted by each institution’s review boards and ethics committees [18]. The study was performed in accordance with the Declaration of Helsinki and its later amendments. Participants provided written informed consent before enrolling in EPIC-IC [18].

### Participants

Participants in EPIC-IC were immunocompromised, were ≥ 12 years old, weighed ≥ 40 kg, and had confirmed SARS-CoV-2 infection (via Reverse Transcription Polymerase Chain Reaction [RT-PCR] or other approved test within 5 days of randomization) and ≥ 1 sign or symptom attributable to COVID-19 on the day of randomization [18]. Signs and symptoms were confirmed using a patient-reported instrument that queried a set of common COVID-19-related symptoms, as recommended in US Food and Drug Administration guidance for COVID-19 trials [34].

Individuals were considered immunocompromised if they met ≥ 1 US Centers of Disease Control and Prevention criteria for moderate or severe IC [18, 35] (Supplementary methods). Individuals were excluded if they needed to be hospitalized or if hospitalization was expected within 24 h of randomization.

Subpopulations with severe IC and non-severe IC were evaluated post hoc [18]. The definition of severe IC was provided by the US Food and Drug Administration based on investigator requests for extended treatment. Participants were considered to have severe IC if they had a hematologic malignancy or had received chimeric antigen receptor T-cell therapy, B-cell‒depleting therapies, or hematopoietic stem cell transplant [18].

### HRQoL in EPIC-IC

As part of the EPIC-IC trial, participants who were ≥ 18 years old at screening were asked to complete several patient-reported outcome (PRO) assessments at baseline (Day 1) and post-baseline visits (Fig. 1). Participants completed PRO assessments using an electronic study diary on an electronic handheld device.

This analysis reports outcomes from the SF-36 and the EQ-5D-5L.

#### SF-36 acute form

The SF-36 assesses a participant’s perceived functional health and well-being over a 1-week recall period [26]. Responses to the 36 items were scored to generate 8 health domain scores: Physical Functioning (10 items), role limitations due to physical health (Role-Physical; 4 items), Bodily Pain (2 items), General Health (5 items), Vitality (4 items), Social Functioning (2 items), role limitations due to emotional problems (Role-Emotional; 3 items), and Mental Health (5 items) [26] (Supplementary methods).

Each domain score was standardized to a z-score using the means and standard deviations (SDs) from 1998 US general population scores [26]. Domain z-scores were then converted to norm-based scores with a mean of 50 and a SD of 10 for reporting.

Physical Component Summary (PCS) and Mental Component Summary (MCS) scores were formed by weighting and aggregating the z-scores from each domain (Supplementary methods) [26]. The PCS and MCS scores were converted to norm-based T-scores (mean = 50, SD = 10) [26]. Norm scores were the mean scores for US general population participants aged 55–64 in the 2005–2006 National Health Measurement Study, calculated as the unweighted mean of male and female mean scores [24].

#### EQ-5D-5L

The EQ-5D-5L is a 5-item survey in which participants describe problems with their health that day in terms of 5 dimensions: Mobility, Self-Care, Usual Activities, Pain/Discomfort, and Anxiety/Depression (Supplementary methods) [28]. Dimension scores were quantified as the proportion of participants reporting “no problems/none” on each dimension. EQ-5D-5L Index values were constructed based on the UK EQ-5D-5L to EQ-5D-3L crosswalk value set from van Hout et al., 2012 [36], as recommended in UK National Institute for Health and Care Excellence (NICE) guidelines at the time of EPIC-IC protocol finalization [37] (Supplementary methods).

### Statistical analysis

Analyses were conducted using SAS Version 9.4. Baseline demographic and clinical characteristics were reported for all randomized participants [18]. HRQoL data were analyzed from the evaluable population of EPIC-IC, defined as participants who received ≥ 1 dose of NMV/r [18]. Analyses were conducted in the overall evaluable sample and in post hoc subpopulations with severe IC and non-severe IC.

Mixed-effects longitudinal models with repeated measures [38] were implemented to assess the effects of 5-day vs. 10-day and 15-day NMV/r on post-baseline SF-36 component summary scores and EQ-5D-5L Index scores (Supplementary methods).

Observed scores and changes from baseline were summarized for each SF-36 domain, SF-36 PCS and MCS, each EQ-5D-5L dimension, and EQ-5D-5L Index at each post-baseline timepoint for each treatment arm and across treatment arms. Changes from baseline were considered statistically significant (*p* < 0.05) if the 95% confidence interval did not include zero.

SF-36 domain, PCS, and MCS scores were described relative to US age-matched (range 55–64 years) general population norm scores [24] and relative to comparator scores of people with cancer (all cancers except skin cancer) [26]. EQ-5D-5L Index scores were described relative to the mean index score in a UK population with various health conditions [39], which used the UK crosswalk value set developed by van Hout et al. [36, 39], and US age-matched (range 55–64 years) general population norm scores [29], which used a US value set [29, 40].

Changes from baseline in SF-36 scores were described relative to anchor-based minimum important difference (MID) thresholds proposed in the SF-36v2 User Manual [26]. For some components, guidelines suggest the use of different MID values for baseline scores < 40 vs. baseline scores ≥ 40 [26]. MID thresholds for the overall sample were selected based on mean baseline scores in the overall sample, and MID thresholds for IC subpopulations were selected based on mean baseline scores for the respective subpopulation. Changes from baseline in EQ-5D-5L Index scores were described relative to anchor-based MID values established in samples with a variety of chronic conditions [41] and in people with cancer [27].

## Results

**Fig. 2:**
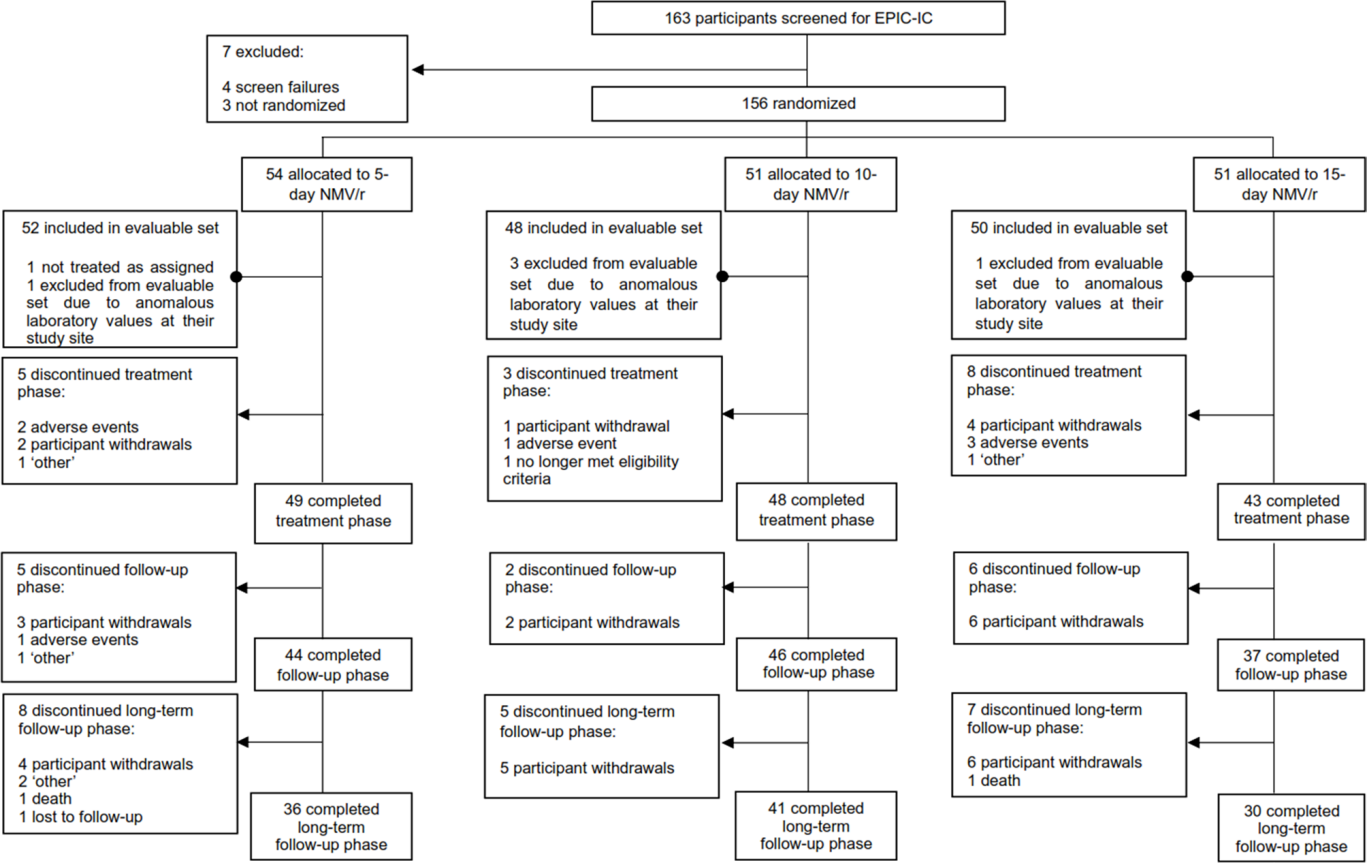
CONSORT flow diagram of participant progress through the EPIC-IC trial. This figure was previously published in Infectious Diseases and Therapy (2025;14:2763–2783) and is reproduced here under the terms of the Creative Commons Attribution-NonCommercial 4.0 International License (CC BY-NC 4.0). Abbreviations: CONSORT, Consolidated Standards of Reporting Trials; NMV/r, nirmatrelvir-ritonavir

EPIC-IC data were collected between August 03, 2022 and November 13, 2023 [18]. A total of 156 participants were randomized, and 155 received ≥ 1 dose of NMV/r (Fig. 2) [18]. SF-36 and EQ-5D-5L data were analyzed from the 150 evaluable participants, 52 of whom were assigned to 5-day NMV/r, 48 to 10-day NMV/r, and 50 to 15-day NMV/r (Fig. 2) [18].

### Baseline demographic and clinical characteristics

The randomized study population spanned 8 countries: Spain (33%), the US (24%), Slovakia (16%), Mexico (15%), Canada (7%), Brazil (3%), Australia (1%), and Bulgaria (1%). Baseline characteristics were generally similar across treatment arms (Table 1). About one-third of participants were characterized as having severe IC (36.5%) (Table 1).

**Table 1:** Demographic and clinical characteristics of randomized participants at baseline (*N* = 156)

Characteristic	All randomized participants ^a^ (*n* = 156)				Severely immunocompromised subpopulation ^a, b^ (*n* = 57)				Non-severely immunocompromised subpopulation ^a, c^ (*n* = 99)			
	5-Day (*n* = 54)	10-Day (*n* = 51)	15-Day (*n* = 51)	Total (*n* = 156)	5-Day (*n* = 20)	10-Day (*n* = 17)	15-Day (*n* = 20)	Total (*n* = 57)	5-Day (*n* = 34)	10-Day (*n* = 34)	15-Day (*n* = 31)	Total (*n* = 99)
**Age in years**, **median (range)**	58.0 (19, 80)	58.0 (16, 82)	58.0 (28, 80)	58.0 (16, 82)	68.5 (19, 77)	66.0 (24, 82)	56.0 (34, 78)	64.0 (19, 82)	54.5 (23, 80)	54.5 (16, 79)	58.0 (28, 80)	57.0 (16, 80)
**Sex**,** female**,** n (%)**	28 (51.9)	30 (58.8)	26 (51.0)	84 (53.8)	9 (45.0)	6 (35.3)	6 (30.0)	21 (36.8)	19 (55.9)	24 (70.6)	20 (64.5)	63 (63.6)
**Race**,** n (%)**												
White	50 (92.6)	47 (92.2)	44 (86.3)	141 (90.4)	17 (85.0)	16 (94.1)	18 (90.0)	51 (89.5)	33 (97.1)	31 (91.2)	26 (83.9)	90 (90.9)
Black or African American	1 (1.9)	0 (0.0)	1 (2.0)	2 (1.3)	1 (5.0)	0 (0.0)	0 (0.0)	1 (1.8)	0 (0.0)	0 (0.0)	1 (3.2)	1 (1.0)
American Indian or Alaska Native	1 (1.9)	3 (5.9)	3 (5.9)	7 (4.5)	0 (0.0)	1 (5.9)	1 (5.0)	2 (3.5)	1 (2.9)	2 (5.9)	2 (6.5)	5 (5.1)
Not reported	2 (3.7)	1 (2.0)	3 (5.9)	6 (3.8)	2 (10.0)	0 (0.0)	1 (5.0)	3 (5.3)	0 (0.0)	1 (2.9)	2 (6.5)	3 (3.0)
**Ethnicity**, **Hispanic or Latino**,** n (%)**												
Yes	37 (68.5)	33 (64.7)	32 (62.7)	102 (65.4)	12 (60.0)	10 (58.8)	11 (55.0)	33 (57.9)	25 (73.5)	23 (67.6)	21 (67.7)	69 (69.7)
Not reported	2 (3.7)	0 (0.0)	0 (0.0)	2 (1.3)	1 (5.0)	0 (0.0)	0 (0.0)	1 (1.8)	1 (2.9)	0 (0.0)	0 (0.0)	1 (1.0)
**BMI**,** kg/m**^**2**^, **median (range)**	27.6 (18, 45)	28.0 (17, 48)	25.0 (16, 46)	27.3 (16, 48)	24.8 (19, 40)	27.3 (17, 31)	23.4 (16, 41)	25.0 (16, 41)	29.8 (18, 45)	28.7 (21, 48)	27.2 (18, 46)	28.3 (18, 48)
**Days since initial SARS-CoV-2 diagnosis**,** median (range)** ^**d**^	1.0 (1, 5)	1.0 (1, 5)	1.0 (1, 4)	1.0 (1, 5)	1.5 (1, 3)	1.0 (1, 5)	1.0 (1, 4)	1.0 (1, 5)	1.0 (1, 5)	1.0 (1, 3)	1.0 (1, 3)	1.0 (1, 5)
**Days since first SARS-CoV-2 symptom**,** median (range)**	3.0 (1, 123)	3.0 (1, 27)	2.0 (1, 174)	3.0 (1, 174)	3.0 (1, 123)	3.0 (1, 8)	3.0 (1, 174)	3.0 (1, 174)	3.0 (1, 5)	3.0 (1, 27)	2.0 (1, 4)	3.0 (1, 27)
**Most recent vaccination against SARS-CoV-2**,** n (%)**												
0–6 months before randomization	7 (13.0)	8 (15.7)	9 (17.6)	24 (15.4)	2 (10.0)	3 (17.6)	5 (25.0)	10 (17.5)	5 (14.7)	5 (14.7)	4 (12.9)	14 (14.1)
> 6 months before randomization	39 (72.2)	37 (72.5)	36 (70.6)	112 (71.8)	16 (80.0)	13 (76.5)	13 (65.0)	42 (73.7)	23 (67.6)	24 (70.6)	23 (74.2)	70 (70.7)
Unvaccinated before randomization	8 (14.8)	6 (11.8)	6 (11.8)	20 (12.8)	2 (10.0)	1 (5.9)	2 (10.0)	5 (8.8)	6 (17.6)	5 (14.7)	4 (12.9)	15 (15.2)
**SARS-CoV-2 NP RNA**,** log**_**10**_ **copies/mL** ^**e**^												
≥ 2, n (%)	49 (90.7)	47 (92.2)	48 (94.1)	144 (92.3)	19 (95.0)	16 (94.1)	18 (90.0)	53 (93.0)	30 (88.2)	31 (91.2)	30 (96.8)	91 (91.9)
≥ 7, n (%)	20 (37.0)	22 (43.1)	28 (54.9)	70 (44.9)	9 (45.0)	5 (29.4)	9 (45.0)	23 (40.4)	11 (32.4)	17 (50.0)	19 (61.3)	47 (47.5)
Median (range)	6.6 (0, 9)	6.8 (0, 9)	7.3 (0, 9)	6.8 (0, 9)	6.9 (4, 9)	6.7 (0, 9)	6.5 (2, 9)	6.8 (0, 9)	6.2 (0, 9)	7.0 (0, 9)	7.5 (0, 9)	7.0 (0, 9)
**Reason for immunocompromise**,** n (%)** ^**f**^												
Immunosuppressant drug therapy	46 (85.2)	46 (90.2)	46 (90.2)	138 (88.5)	13 (65.0)	16 (94.1)	17 (85.0)	46 (80.7)	33 (97.1)	30 (88.2)	29 (93.5)	92 (92.9)
Hematologic malignancy	19 (35.2)	15 (29.4)	20 (39.2)	54 (34.6)	19 (95.0)	15 (88.2)	20 (100.0)	54 (94.7)	0 (0.0)	0 (0.0)	0 (0.0)	0 (0.0)
Immunocompromise based solely on corticosteroids or TNF blockers	11 (20.4)	9 (17.6)	10 (19.6)	30 (19.2)	0 (0.0)	0 (0.0)	0 (0.0)	0 (0.0)	11 (32.4)	9 (26.5)	10 (32.3)	30 (30.3)
CAR-T-cell therapy/ hematopoietic stem cell transplant	7 (13.0)	7 (13.7)	6 (11.8)	20 (12.8)	7 (35.0)	7 (41.2)	6 (30.0)	20 (35.1)	0 (0.0)	0 (0.0)	0 (0.0)	0 (0.0)
Solid tumor	8 (14.8)	5 (9.8)	6 (11.8)	19 (12.2)	0 (0.0)	0 (0.0)	0 (0.0)	0 (0.0)	8 (23.5)	6 (17.6)	6 (19.4)	20 (20.2)
Moderate or severe primary immunodeficiency/HIV infection CDC Group III < 200 CD4 count	2 (3.7)	1 (2.0)	2 (3.9)	5 (3.2)	0 (0.0)	0 (0.0)	1 (5.0)	1 (1.8)	1 (2.9)	1 (2.9)	1 (3.2)	3 (3.0)
Solid organ transplant	1 (1.9)	1 (2.0)	0 (0.0)	2 (1.3)	0 (0.0)	0 (0.0)	0 (0.0)	0 (0.0)	1 (2.9)	1 (2.9)	0 (0.0)	2 (2.0)
Not meeting any CRF category	0 (0.0)	2 (3.9)	1 (2.0)	3 (1.9)	0 (0.0)	0 (0.0)	0 (0.0)	0 (0.0)	0 (0.0)	1 (2.9)	1 (3.2)	2 (2.0)

^a^ Demographic and clinical characteristics are reported at baseline for all 156 randomized participants. Analyses were conducted on the evaluable set, which included 150 of the 156 randomized participants (57 of the 57 severely immunocompromised subpopulation and 93 of the 99 non-severely immunocompromised subpopulation)

^b^ Severely immunocompromised participants were those who met ≥ 1 of the following criteria: hematologic malignancy; history of CAR-T-cell therapy, B-cell‒depleting therapies, or hematopoietic stem cell transplant

^c^ Non-severe IC participants were those who did not meet the criteria for severe IC

^d^ Pertains to current SARS-CoV-2 infection

^e^ In EPIC-IC, the LLOQ for SARS-CoV-2 NP RNA was defined as 2.0 log_10_ copies/mL

^f^ Not mutually exclusive

Abbreviations: BMI, body mass index; CAR-T-cell, chimeric antigen receptor T-cell; CDC, Centers for Disease Control and Prevention; CD4, cluster of differentiation 4; CRF, case report form; HIV, human immunodeficiency virus; IC, immunocompromise; LLOQ, lower limit of quantification; NMV/r, nirmatrelvir-ritonavir; NP, nasopharyngeal; RNA, ribonucleic acid; SARS-CoV-2, severe acute respiratory syndrome coronavirus 2; SD, standard deviation; TNF, tumor necrosis factor

### SF-36 completion and outcomes

The SF-36 was completed by 51% of participants at baseline and by 83–90% of participants across post-baseline timepoints (Supplementary Table 1; Supplementary results).

### SF-36 domain scores

#### Overall sample

For all domains, baseline scores appeared to be similar across treatment arms (Supplementary Fig. 1; Supplementary Tables 2–9). Across arms, mean baseline domain scores were lowest (i.e., indicating worse HRQoL) in Social Functioning (35.7, norm-based with a mean of 50 and SD of 10) and Role-Physical (35.7) domains and highest in the Mental Health (44.1) domain (Supplementary Tables 2–9). For each domain, mean baseline scores in the overall sample were substantially lower (i.e., worse) than US general population norm scores for ages 55–64 [24], population norm scores for ages 55–64 provided in the SF-36 User Manual [26], and comparator scores for people with cancer (all cancers except skin cancer) [26].

Post-baseline improvements in the 5-day arm generally appeared similar to those in 10-day and 15-day arms (Supplementary Fig. 1; Supplementary Tables 2–9). Consequently, scores are summarized below across treatment arms.

**Fig. 3:**
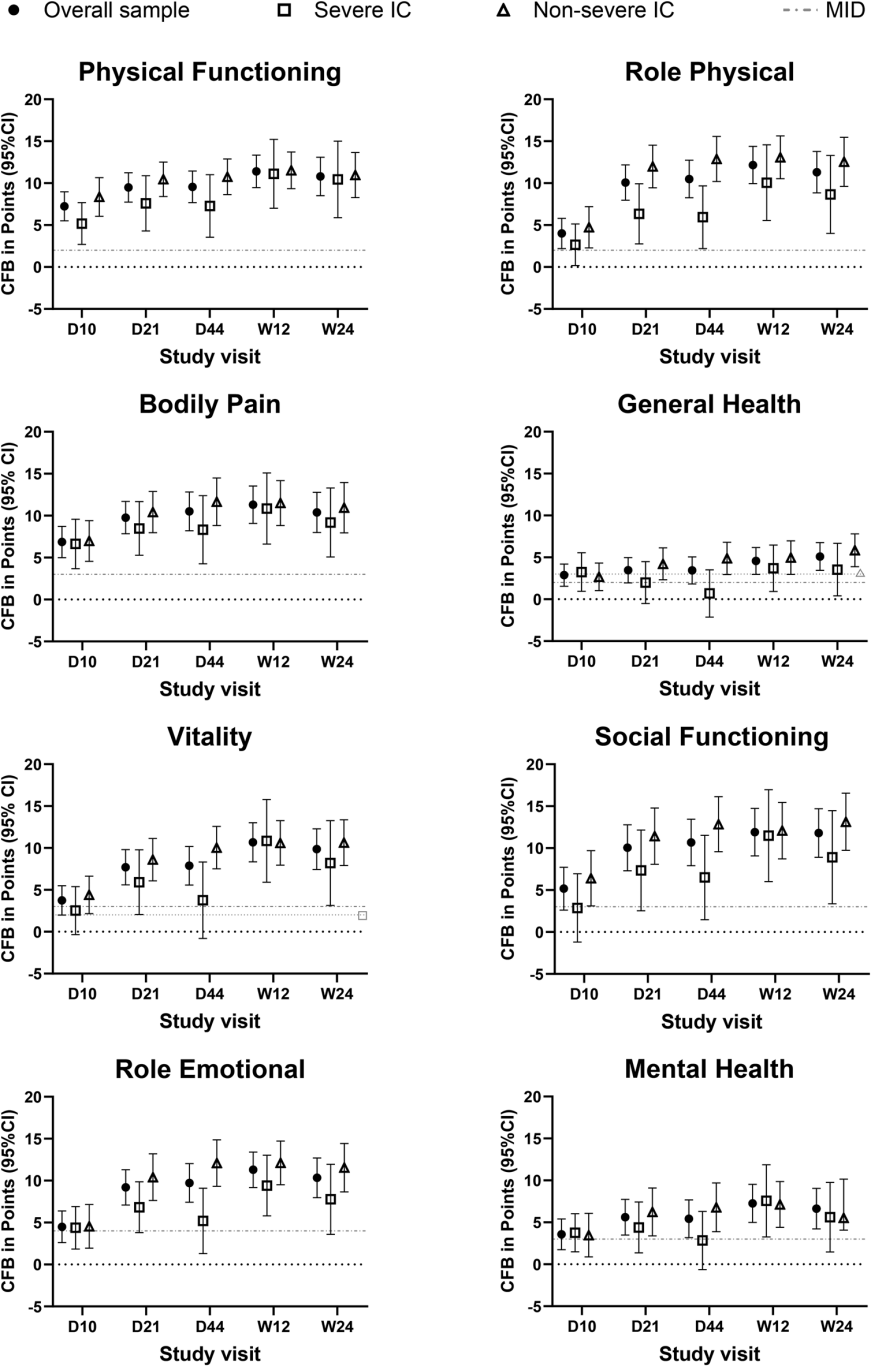
Changes from baseline in SF-36 component scores. Mean (95% CI) change from baseline in points in the overall sample (*N* = 150; filled circles), severe IC subpopulation (*n* = 57; open squares), and non-severe IC subpopulation (*n* = 93; open triangles) for each SF-36 component at each post-baseline visit where the SF-36 was administered. SF-36 component scores were calculated using norm-based scoring (mean = 50, SD = 10), with higher scores representing better HRQoL. Grey lines depict guideline-suggested MID values, reflecting points improved using norm-based scoring [26]. For some components, guidelines suggest the use of different MID values for baseline scores < 40 vs. baseline scores ≥ 40 [26]; MID values shown are based on the mean baseline score in the overall sample. In two cases, different MID values were suggested for IC subpopulations than for those in the overall sample: the MID value suggested for General Health in the non-severe IC subpopulation was 3, and the MID value suggested for Vitality in the severe IC subpopulation was 2. Abbreviations: CFB, change from baseline; CI, confidence interval; D, day; IC, immunocompromise; MID, minimum important difference; SF-36, 36-Item Short Form Health Survey – acute form; W, week

For all domains, scores were significantly improved from baseline at each post-baseline visit (all *p* < 0.05; Fig. 3; Supplementary Tables 2–9). Mean improvements at each post-baseline visit were numerically greater than published MID values proposed in the SF-36 User Manual for comparing group mean differences (not specific to any population); MID values ranged from 2 to 4 points, using norm-based scoring [26] (Fig. 3).

At Day 44, mean improvements from baseline were greatest in Social Functioning (10.7 points; 29.9% increase) and Role-Physical (10.5 points; 29.4% increase) and smallest in General Health (3.4 points; 8.8% increase) and Mental Health (5.4 points; 12.3% increase) (Fig. 3; Supplementary Tables 2–9). Improvements followed the same order across domains at Week 24 (Supplementary Tables 2–9).

#### Severe IC and non-severe IC subpopulations

Baseline SF-36 domain scores were generally similar across treatment arms in the non-severe IC subpopulation, whereas several numeric differences were observed across treatment arms in the severe IC subpopulation (Supplementary Fig. 1; Supplementary results). For simplicity, scores below are reported across treatment arms. Baseline scores were generally similar between severe vs. non-severe IC subpopulations, except the mean General Health score was numerically lower (i.e., worse) in those with severe IC (35.8 vs. 41.2).

In the non-severe IC subpopulation, all domain scores increased (i.e., improved) significantly from baseline at each post-baseline visit (each *p* < 0.05; Fig. 3), while several improvements in the severe IC subpopulation did not reach statistical significance (Fig. 3; Supplementary results).

The subpopulation with severe IC had a trend of less improvement from baseline than the non-severe IC subpopulation in the Role-Physical and Role-Emotional domains from Day 21 through Week 24 (Fig. 3). Improvements were similar between subpopulations in other domains.

Mean improvements from baseline were numerically higher than MID values (which ranged from 2 to 4 points, using norm-based scoring) for both subpopulations, except for 4 mean domain scores in the severe IC subpopulation (General Health at Day 21 and Day 44, Social Functioning at Day 10, and Mental Health at Day 44) and 1 mean domain score in the non-severe IC subpopulation (General Health at Day 10) (Fig. 3).

### SF-36 PCS and MCS scores

#### Overall sample

Baseline PCS and MCS scores appeared similar across treatment arms (Supplementary Figs. 3, 5; Supplementary Tables 10–11). Mean baseline PCS and MCS scores in the overall sample (PCS: 37.88; MCS: 41.51; norm-based with a mean of 50 and SD of 10) and in each arm (Supplementary Tables 10–11) were substantially below (i.e., worse than) age-matched general population norm scores (PCS: 47.55; MCS: 53.87) [24] and norm scores of people with cancer (all types except skin; PCS: 41.11; MCS: 47.66) [26].

For all post-baseline timepoints, PCS and MCS scores in the 5-day arm were similar to those in the extended treatment arms (all *p* > 0.05) (Supplementary Tables 10–11; Supplementary Figs. 3, 5). Consequently, PCS and MCS scores below are described across arms.

The PCS scores increased (i.e., improved) substantially through Day 44 and remained stable through Week 24, with mean post-baseline scores approaching age-matched general population scores at Day 44 and exceeding age-matched scores from Week 12 (Supplementary Fig. 3). The MCS scores increased through Day 21 and remained stable and slightly below age-matched general population scores through Week 24 (Supplementary Fig. 5.) At Week 24, PCS scores had improved by 10 points from baseline and MCS scores by 8 points from baseline.

**Fig. 4:**
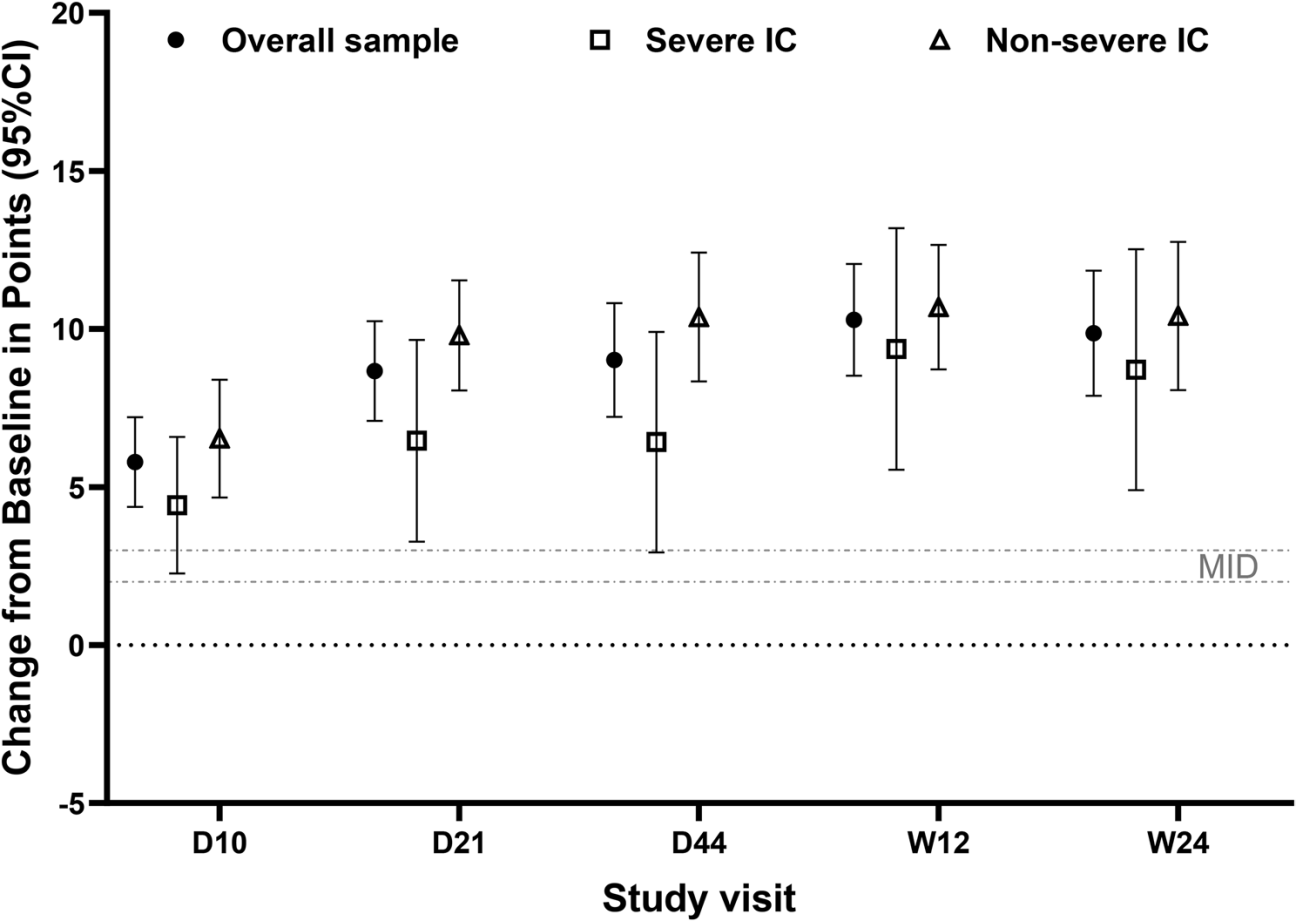
Changes from baseline in SF-36 Physical Component Summary scores. Mean (95% CI) change from baseline in points in the overall sample (*N* = 150; filled circles), severe IC subpopulation (*n* = 57; open squares), and non-severe IC subpopulation (*n* = 93; open triangles) for the SF-36 Physical Component Summary score at each post-baseline visit where the SF-36 was administered. SF-36 Physical Component Summary scores are norm-based (mean = 50, SD = 10), with higher scores representing better HRQoL. Grey lines depict the MID range of 2–3 points for the Physical Component Summary score [26]. Abbreviations: CI, confidence interval; D, day; IC, immunocompromise; MID, minimum important difference; SF-36, 36-Item Short Form Health Survey – acute form; W, week

**Fig. 5:**
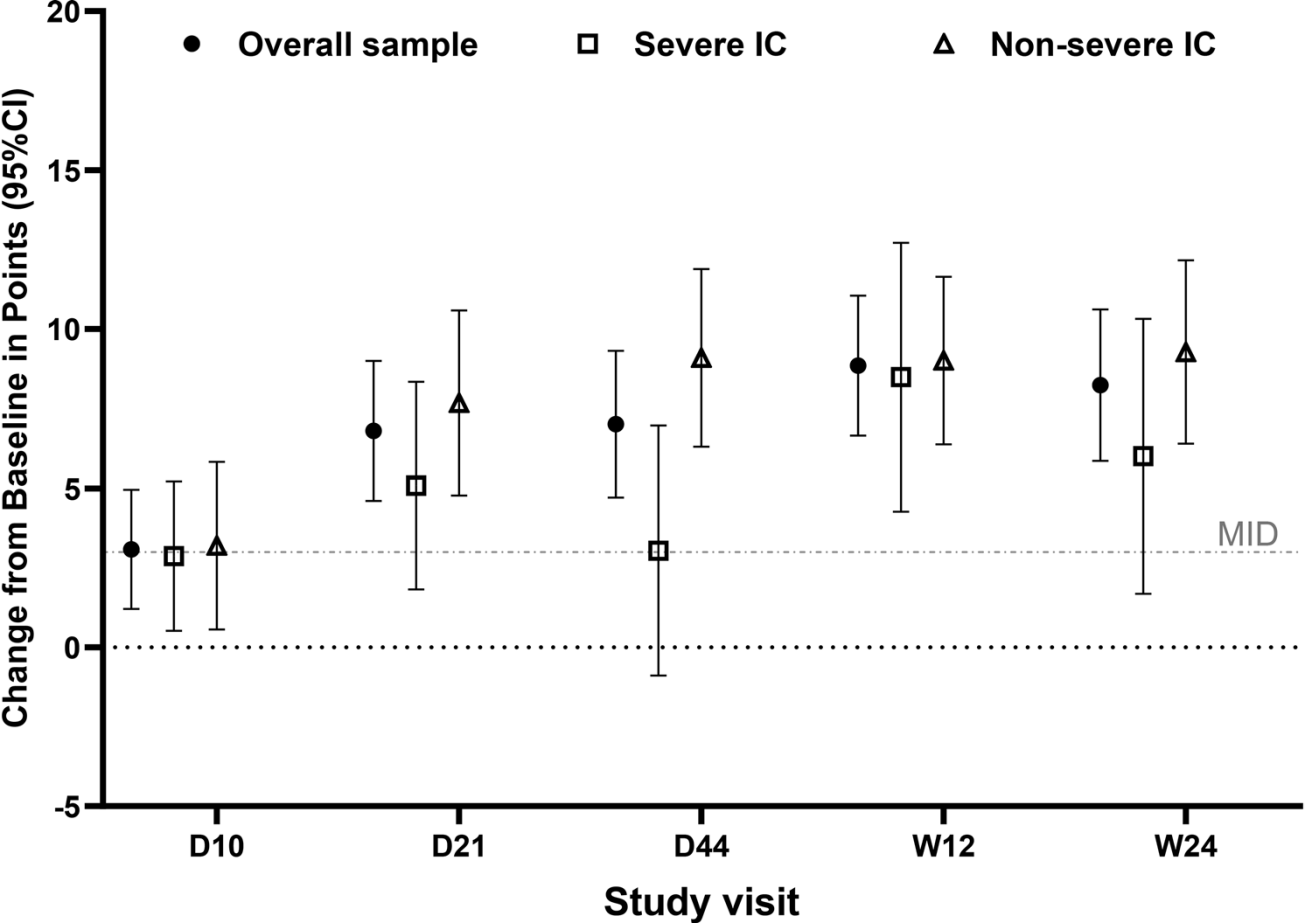
Changes from baseline in SF-36 Mental Component Summary scores. Mean (95% CI) change from baseline in points in the overall sample (*N* = 150; filled circles), severe IC subpopulation (*n* = 57; open squares), and non-severe IC subpopulation (*n* = 93; open triangles) for the SF-36 Mental Component Summary score at each post-baseline visit where the SF-36 was administered. Grey lines depict the MID value of 3 for the Mental Component Summary score [26]. Abbreviations: CI, confidence interval; D, day; IC, immunocompromise; MID, minimum important difference; SF-36, 36-Item Short Form Health Survey – acute form; W, week

At each post-baseline timepoint, PCS and MCS improvements from baseline were statistically significant (all *p* < 0.05) (Figs. 4 and 5; Supplementary Tables 10–11). For both PCS and MCS, each mean post-baseline improvement was numerically greater than the recommended group-level MID of 2–3 points [26], indicating substantial improvement (Figs. 4 and 5; Supplementary Tables 10–11).

#### Severe IC and non-severe IC subpopulations

In the severe IC subpopulation, baseline PCS score in the 5-day arm (32.9) was numerically lower (i.e., worse) than those in the 10-day arm (38.4) and 15-day arm (37.2), and the mean baseline MCS score was numerically higher (i.e., better) in the 5-day arm (43.5) than in the 15-day arm (39.3) (Supplementary Figs. 4, 6). In the non-severe IC subpopulation, no substantial baseline PCS differences were observed between arms, whereas mean baseline MCS scores were notably higher (i.e., better) in the 5-day arm (44.1) than in the 10-day arm (39.4) (Supplementary Figs. 4, 6). Across arms, mean baseline PCS scores were numerically lower in the severe IC subpopulation (36.0) than in the non-severe IC subpopulation (39.0). Mean baseline MCS scores were similar between severe IC (41.3) and non-severe IC (41.6) subpopulations.

Day 10 PCS scores in the severe IC subpopulation were significantly higher (i.e., better) in the 10-day vs. 5-day arm (least-squares mean [standard error], 43.0 [3.4] vs. 35.8 [3.6]; *p* = 0.03). No other statistically significant differences were observed between treatment arms for PCS or MCS (each *p* > 0.05). Results below are summarized across treatment arms.

Mean PCS and MCS scores improved across follow-up in both subpopulations, although numerically less improvement was observed in the severe IC subpopulation (Figs. 4 and 5). Improvements from baseline in PCS and MCS were statistically significant at each post-baseline visit for each subpopulation (each *p* < 0.05), except for MCS in the severe IC subpopulation at Day 44 (*p* > 0.05) (Figs. 4 and 5). Mean post-baseline improvements in PCS and MCS scores were numerically greater than the recommended group-level MIDs of 2–3 points at all timepoints, except for mean improvement in MCS at Day 10 in the severe IC subpopulation (2.9) [26] (Figs. 4 and 5).

The mean PCS scores for each treatment arm in the non-severe IC subpopulation surpassed the general population norm score by Day 21 and remained above (i.e., better than) this norm score throughout long-term follow-up, whereas mean PCS scores for each arm in the severe IC subpopulation approached but remained below (i.e., worse than) the general population norm (Supplementary Fig. 4). In contrast, mean MCS scores remained below (i.e., worse than) population norm scores throughout long-term follow-up in both the severe IC and non-severe IC subpopulations (Supplementary Fig. 6).

### EQ-5D-5L completion and outcomes

Nearly all participants (93%) completed the EQ-5D-5L at baseline, and post-baseline completion rates ranged from 83 to 89% across timepoints (Supplementary Table 12; Supplementary results).

### EQ-5D-5L dimension scores

#### Overall sample

**Fig. 6:**
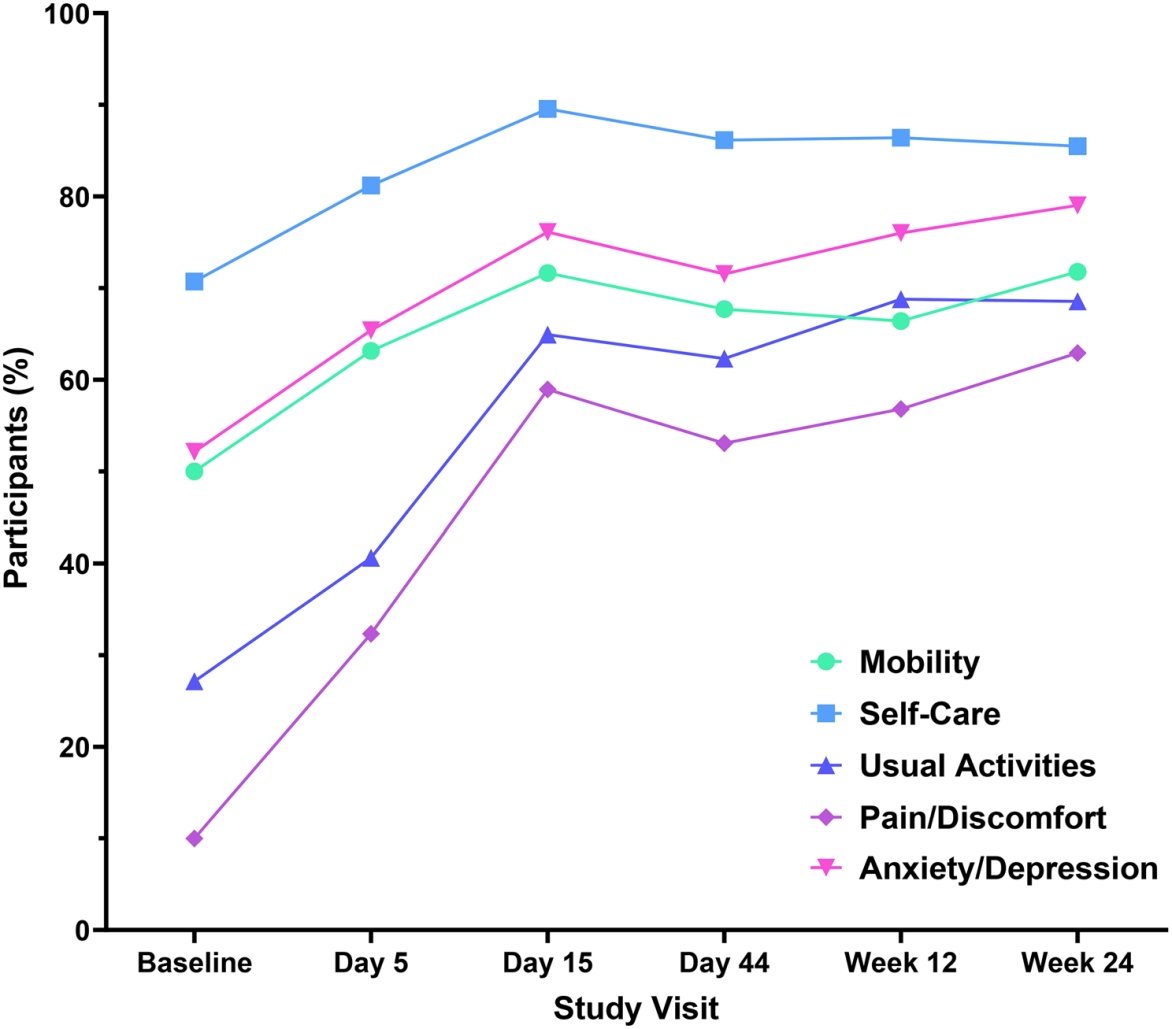
Participants reporting “no problems/none” in EQ-5D-5L dimensions. Percentage of participants in the overall sample (*N* = 150) reporting “no problems/none” in EQ-5D-5L dimensions at each study visit

At baseline, the proportions of participants reporting problems on the EQ-5D-5L varied widely across dimensions (Fig. 6; Supplementary Tables 13–17). Substantially fewer participants in the 5-day arm reported baseline problems with anxiety/depression and self-care than did those in other arms (Supplementary Fig. 7). For simplicity, proportions are summarized below for the overall sample.

At baseline, most participants reported at least some pain/discomfort (90%) or at least some problems with maintaining their usual activities (73%), and about half reported problems with mobility (50%) or anxiety/depression (48%) (Fig. 6). Only 29% reported problems with self-care (Fig. 6).

In all dimensions, the proportions of participants reporting problems decreased substantially from baseline through Day 15, by 49 percentage points for pain/discomfort, 38 percentage points for usual activities, 22 percentage points for mobility, 24 percentage points for anxiety/depression, and 19 perecentage points for self-care (Fig. 6). Proportions then remained stable through Week 24 (Fig. 6).

#### Severe IC and non-severe IC subpopulations

For most EQ-5D-5L dimensions, the proportions of participants reporting problems differed substantially across treatment arms in the severe IC subpopulation but were generally similar across arms in the non-severe IC subpopulation (Supplementary Fig. 8). Results are described below across treatment arms.

**Fig. 7:**
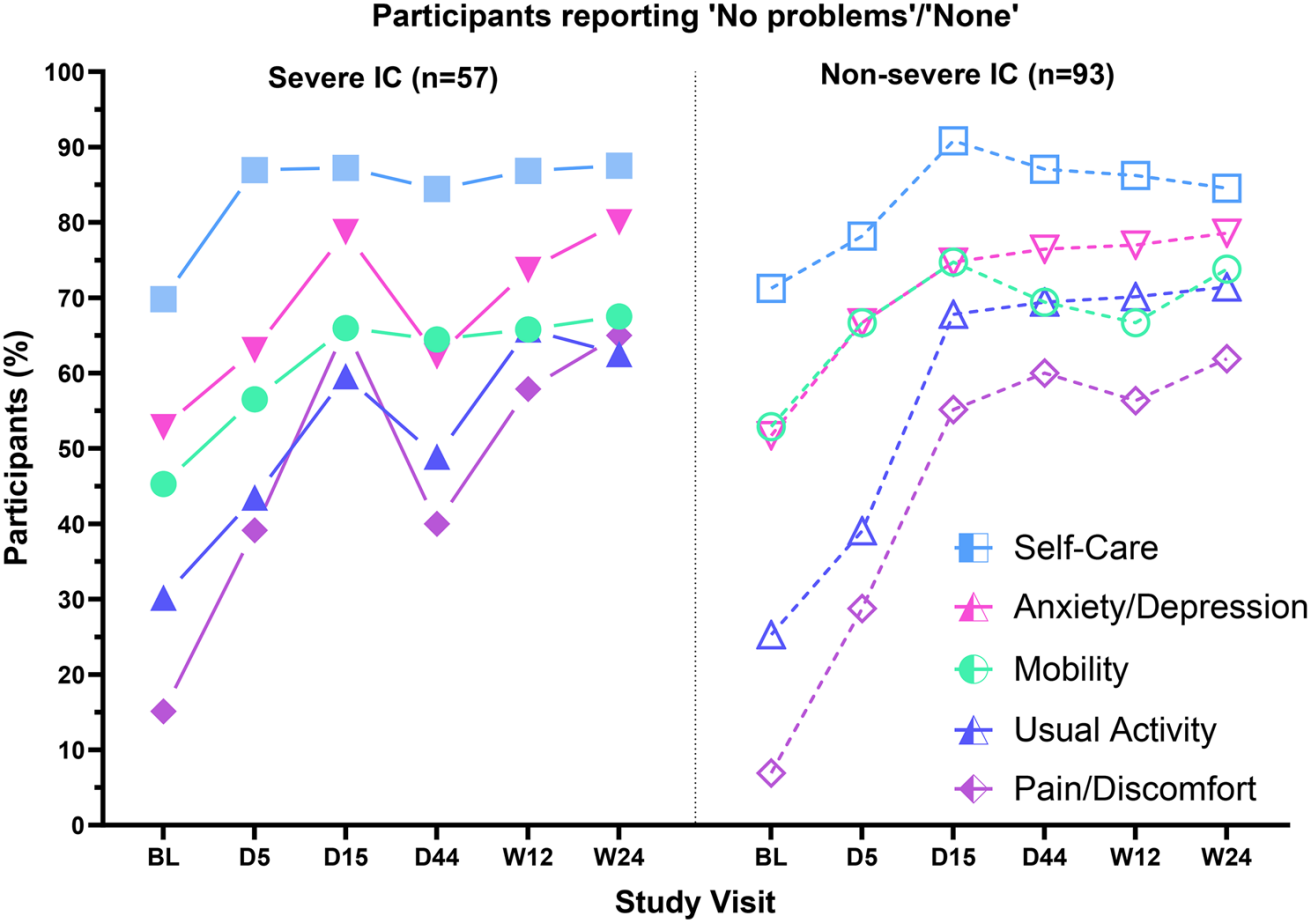
Participants with severe IC vs. non-severe IC reporting “no problems/none” in EQ-5D-5L domains. Percentage of participants with severe IC (left panel) and non-severe IC (right panel) across treatment arms reporting “no problems/none” in EQ-5D-5L domains at each study visit. Abbreviations: BL, baseline; D, Day; IC, immunocompromise; W, week

At baseline, slightly more participants with severe IC than non-severe IC reported problems with mobility, and slightly more participants with non-severe IC than severe IC reported pain/discomfort and problems with usual activities (Fig. 7).

The proportion of participants reporting problems decreased from baseline at Day 5 and Day 15 for all dimensions in both subpopulations (Fig. 7). In the subpopulation with non-severe IC, the proportions of participants with problems remained stable in all dimensions from Day 15 through Week 24. An increased proportion of participants in the severe IC subpopulation reported problems with pain/discomfort, anxiety/depression, and usual activities at Day 44, suggesting a reversal in the pattern of improvement in these domains. By Week 12 and at Week 24, the proportion of severe IC participants reporting problems in these dimensions improved, ending at around the peak levels reached at Day 15.

No interactions were observed between IC severity and treatment group (Supplementary Fig. 8), except that a relatively high proportion of participants with severe IC in the 10-day and 15-day arms reported problems at Day 44.

### EQ-5D-5L index score

#### Overall sample

Baseline EQ-5D-5L Index scores appeared similar across treatment arms (Supplementary Fig. 9; Supplementary Table 18). Mean baseline scores in the overall sample (0.65; scale: −0.594–1.000 [42], with higher scores reflecting better HRQoL) and each treatment arm (0.63–0.67) were within the range of the mean index score of a UK population with various health conditions (0.65) [39] and lower (i.e., worse) than age-matched US population norms (0.815 face-to face; 0.781 online) [29].

For all post-baseline timepoints, scores in the 5-day arm were similar to those in the 10-day and 15-day arms (all *p* > 0.05; Supplementary Fig. 9; Supplementary Table 18). Results are described below across treatment arms.

**Fig. 8:**
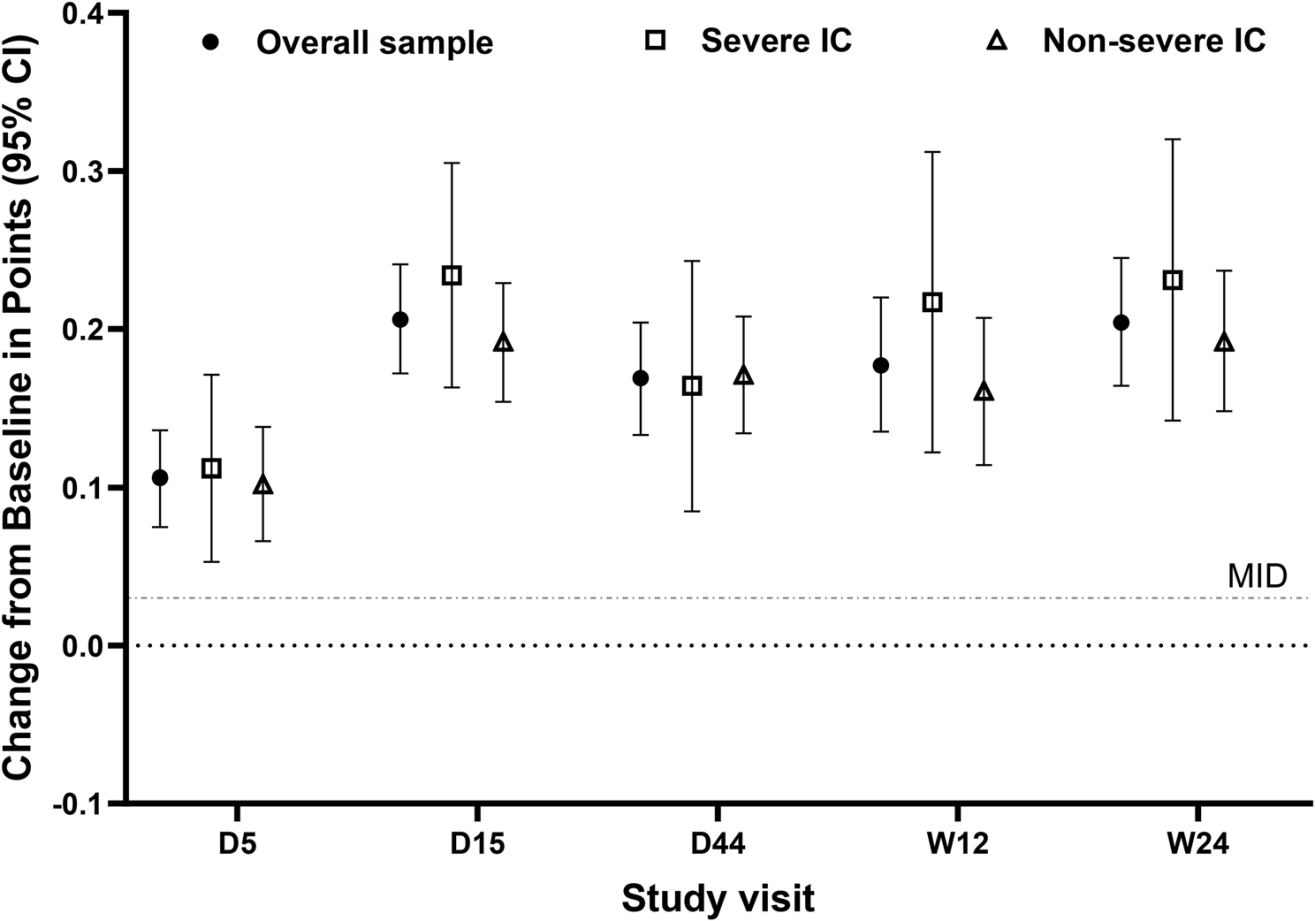
Changes from baseline in EQ-5D-5L Index scores. Mean (95% CI) change from baseline in points in the overall sample (*N* = 150; filled circles), severe IC subpopulation (*n* = 57; open squares), and non-severe IC subpopulation (*n* = 93; open triangles) for the EQ-5D-5L Index score at each post-baseline visit where the EQ-5D-5L was administered. The grey dotted line depicts the estimated MID value (0.03) for the EQ-5D-5L Index in a sample with cancer [27]. Abbreviations: CI, confidence interval; D, Day; IC, immunocompromise; MID, minimum important difference; W, week

Scores were significantly improved from baseline at each post-baseline visit (all *p* < 0.05) (Fig. 8; Supplementary Table 18). Mean scores rose to a peak of 0.85 at Day 15, and the mean increase from baseline at Day 15 among participants with baseline data was 0.21 points. Mean scores remained stable through Week 24, where change from baseline was 0.20 points.

At each post-baseline timepoint, increases from baseline substantially surpassed MID values established in samples with cancer (MID estimate, 0.03 points) [27] and a variety of chronic conditions (mean [SD] MID, 0.05 [0.03] points) [41] (Fig. 8).

#### Severe IC and non-severe IC subpopulations

In the severe IC subpopulation, mean baseline scores were numerically lower (i.e., worse) in the 15-day arm (0.57) than in the 5-day (0.62) or 10-day (0.64) arms (Supplementary Fig. 10). In the non-severe IC subpopulation, scores appeared similar across arms (Supplementary Fig. 10). Results below are described across treatment arms.

Baseline scores were numerically lower (i.e., worse) in the subpopulation with severe IC (mean [SD], 0.61 [0.25]) than in the subpopulation with non-severe IC (0.68 [0.17]) (Supplementary Fig. 10).

At all post-baseline timepoints, scores in the 5-day arm were similar to those in the 10-day and 15-day arms for both subpopulations (all *p* > 0.05).

Scores improved significantly from baseline to each post-baseline visit for both subpopulations (all *p* < 0.05; Fig. 8) and surpassed MID values established in patients with chronic conditions (mean, 0.05; SD, 0.03) [41] and in patients with cancer (0.03) [27] (Fig. 8).

## Discussion

Limited information is available on HRQoL in people with IC during COVID-19 illness and recovery. SF-36 and EQ-5D-5L data from the EPIC-IC trial show that immunocompromised adults with mild–moderate COVID-19 experienced physical, social, mental, and daily living problems during early COVID-19 illness. All HRQoL outcomes improved meaningfully during and following treatment with NMV/r, with improvements sustained through long-term follow-up. Improvements were similar across NMV/r treatment durations. Although HRQoL improved substantially in both severe IC and non-severe IC subpopulations, some aspects of HRQoL improved more slowly or to a lesser degree in those with severe IC.

Baseline HRQoL varied widely across SF-36 domains and EQ-5D-5L dimensions. Across SF-36 domains, scores were relatively worse for Role-Physical and Social Functioning and relatively better for Mental Health. This domain ordering is similar to that in a sample with cancer and without COVID-19 [26], except that Social Functioning scores were relatively lower (i.e., worse) in EPIC-IC [26]. Notably, mean baseline SF-36 scores in our sample were numerically lower (i.e., worse) than those in the cancer norm sample across all domains. This may reflect the negative impact of mild–moderate COVID-19 on HRQoL [8] or differences between the samples (e.g., in overall health status). Although cancer was the most common immunocompromising condition in our sample, over half of EPIC-IC participants had different immunocompromising conditions or medication use. Domain ordering in EPIC-IC was different from that in a China-based elderly sample hospitalized with COVID-19, with domain ordering suggesting relatively better Physical Functioning and relatively worse Role-Physical scores in EPIC-IC participants than in the China-based sample, although all domain scores were worse in EPIC-IC [43]; these differences could be attributed to IC, age, cultural differences related to the different geographic settings, or COVID-19 severity.

EQ-5D-5L dimension scores suggest that participants’ most common issues at baseline were pain/discomfort and problems with usual activities. The distribution of problems across dimensions was similar to that observed in the multinational EPIC-HR trial, which assessed 5-day NMV/r vs. placebo in a broad group of participants with mild–moderate COVID-19 who were at high risk for developing severe COVID-19, except that mobility problems were relatively more common in EPIC-IC [44]. In each dimension, problems were reported by a substantially greater proportion of EPIC-IC participants than EPIC-HR participants [44]. This may reflect the older age of our sample [32] or could provide evidence for worse HRQoL in immunocompromised vs. broad high-risk populations.

Baseline mean EQ-5D-5L Index scores (0.65) were similar to those in a UK-based general population sample with mild–moderate COVID-19 (0.62) [7] but substantially lower (i.e., worse) than those in a US-based general population sample with symptomatic COVID-19 (0.81) [45]. The lower scores in EPIC-IC may reflect impacts of underlying health conditions, older age [29, 31], or cultural influences driving country-specific EQ-5D-5L norms [31]. Baseline EQ-5D-5L scores were also lower than those in multinational meta-analyses of cancer patients (0.75) [32] and HIV patients (0.84) without COVID-19 [32] and age-matched US general population norms (0.815 face-to-face; 0.781 online) [29], which may highlight the detrimental impact of early COVID-19 illness on HRQoL across diverse clinical contexts. Overall, immunocompromised adults with mild–moderate COVID-19 experienced worse HRQoL than adults in the general population or with other chronic health conditions and similar or worse HRQoL than general population samples with mild–moderate COVID-19.

HRQoL was significantly improved from baseline at each post-baseline timepoint. Importantly, improvement at each timepoint surpassed published MID thresholds [26, 27, 41]. Together, these data suggest that baseline HRQoL reflected some impact of COVID-19 and that participants experienced meaningful improvements within 5–10 days from baseline. Specific MID thresholds have not been published for immunocompromised populations or for acute COVID-19; when available, these would provide added context for HRQoL improvements [41].

Improvements in EQ-5D-5L outcomes generally peaked by Day 15, aligning with the approximately 13–16 day timing of overall symptoms resolution in EPIC-IC [17], whereas SF-36 outcomes continued improving through Week 12. The continued improvements in SF-36 outcomes appear to have been driven by slower, less-pronounced improvements in the severe IC subpopulation. This pattern may reflect the greater nuance or the longer recall window of the SF-36 (relative to the EQ-5D-5L or the questionnaire used to assess symptoms resolution) [17, 26, 46, 47], which might have allowed the SF-36 to capture subtle or infrequent longer-term detriments to HRQoL occurring in participants with severe IC.

Mean EQ-5D-5L Index scores matched the norm score of a UK-based sample with various health conditions (0.65) [39] at baseline and improved to surpass these UK norms from Day 5, and then improved further to reach or surpass cancer norms [26] by Day 15. SF-36 scores suggest that physical aspects of HRQoL improved to approach general population levels by Day 44 [24], while mental aspects approached but remained below (i.e., worse than) general population levels throughout the study [24]. Similarly, HRQoL data in general population samples with mild–moderate COVID-19 show improvement to pre-COVID-19 EQ-5D-5L Index scores “post-COVID-19” [7] and suggest more nuanced recovery of SF-36 scores [8]. In a Netherlands-based general population sample, those with mild-severity COVID-19 recovered to norm levels on all SF-36 domains after 12 months, whereas those with moderate-severity COVID-19 had significant deficits in 6 of 8 domains [8].

HRQoL improvement did not differ by NMV/r treatment duration, except that SF-36 PCS scores in the severe IC subpopulation were significantly worse at Day 10 in those treated with 5-day vs. 10-day NMV/r (*p* = 0.03). This finding is similar to the slower global symptoms resolution in severely immunocompromised participants treated with 5-day vs. 10-day NMV/r (*p* = 0.03) [17] and the nominally lower likelihood of sustained viral clearance in severely immunocompromised participants treated with 5-day vs. extended NMV/r [18]. Additional studies are needed to understand whether extended NMV/r provides a genuine benefit to physical aspects of HRQoL recovery in people with severe IC.

This study is the first to report SF-36 or EQ-5D-5L outcomes in an immunocompromised sample with mild–moderate COVID-19. One strength of this study is that HRQoL was reported at multiple timepoints, from the first days of COVID-19 symptoms to 24 weeks. Further, PRO measures were completed at the relevant timepoint, rather than retrospectively. Another strength is that the SF-36 and EQ-5D-5L are multidimensional, well-established, validated HRQoL measures [24, 26–32, 36, 39, 41, 47], thus providing rich information on HRQoL that can be considered in the context of samples with similar baseline illness [26, 27, 32] or samples with COVID-19 [1–7, 44, 45]. Finally, our sample was carefully vetted and confirmed to have mild–moderate COVID-19 illness and immunocompromise as part of the EPIC-IC trial [18].

The findings from this study can be used to inform expectations for HRQoL recovery, highlighting which aspects of HRQoL are most likely to be impacted and when each aspect might be expected to improve. Clinical monitoring can be guided to focus on aspects of HRQoL that show the greatest detriments or are most amenable to improvement. In addition, findings from this study complement viral load and symptom findings from EPIC-IC to show no further benefit of extended NMV/r regimens over a 5-day regimen, aside from limited evidence of potential added benefit in individuals with severe IC [17, 18].

A limitation of this study is that only about half of participants completed the SF-36 at baseline. Consequently, SF-36 outcomes may not fully reflect HRQoL in participants unable or unwilling to complete the SF-36 during early acute COVID-19 illness. If only those with milder illness completed the SF-36 at baseline, reported findings might underestimate the impact of COVID-19 on SF-36-reported HRQoL. Interestingly, baseline SF-36 completion rates were numerically higher among those with severe IC (58%) vs. non-severe IC (46%), whereas baseline EQ-5D-5L completion rates were similar across IC severity subpopulations (93% vs. 94%). Another limitation of this study is that EPIC-IC did not include an untreated control group [18], as it would have been unethical to withhold an available, guideline-recommended treatment [19–21] from this vulnerable population. Improvements from baseline may therefore reflect the natural course of recovery from COVID-19 in addition to any treatment-related effects. Study results in subgroups with severe vs. non-severe IC should be interpreted with particular caution, as these analyses were conducted post hoc. Moreover, demographic differences between the severe IC and non-severe IC subpopulations may confound interpretation of differences between IC severity subgroups: the severe IC subpopulation was older and had a higher proportion of male participants than the non-severe IC subpopulation, and both older age and male sex have been associated with negative COVID-19 outcomes [48]. Finally, baseline HRQoL and recovery may differ in populations with different reasons for IC [49, 50].

## Conclusions

Immunocompromised adults from EPIC-IC experienced broad HRQoL problems during mild–moderate COVID-19 illness. Each aspect of HRQoL improved during and after treatment with NMV/r, with improvements sustained over 24 weeks. Recovery patterns were similar across those treated with 5-day, 10-day, or 15-day NMV/r. Individuals with severe IC showed slower or less improvement in some aspects of HRQoL, highlighting the need for larger, prospective studies to confirm differences in HRQoL recovery across IC subpopulations.

## Supplementary Information

Below is the link to the electronic supplementary material.

Supplementary Material 1

## Data Availability

Upon request, and subject to review, Pfizer will provide the data that support the findings of this study. Subject to certain criteria, conditions and exceptions, Pfizer may also provide access to the related individual de-identified participant data. See https://www.pfizer.com/science/clinical-trials/trial-data-and-results for more information.

## Abbreviations

CAR-T-cell: Chimeric Antigen Receptor T-cell
CIOMS: Council for International Organizations of Medical Sciences
COVID-19: Coronavirus Disease 2019
EQ-5D-5L: 5-Level EQ-5D
ESCMID: European Society of Clinical Microbiology and Infectious Diseases
HIV: Human Immunodeficiency Virus
HRQoL: Health-Related Quality of Life
IC: Immunocompromise
ICH: International Council for Harmonisation
ISPOR: International Society for Pharmacoeconomics and Outcomes Research
MCS: Mental Component Summary
MID: Minimum Important Difference
NMV/r: Nirmatrelvir-ritonavir
PCS: Physical Component Summary
PRO: Patient-Reported Outcome
RNA: Ribonucleic Acid
RT-PCR: Reverse Transcription Polymerase Chain Reaction
SARS-CoV-2: Severe Acute Respiratory Syndrome Coronavirus 2
SD: Standard Deviation
SF-36: 36-Item Short Form Health Survey
