# Supplementary material for: Health-related quality of life in immunocompromised adults with mild–moderate COVID-19 treated with nirmatrelvir-ritonavir: results from the randomized, double-blinded EPIC-IC trial
Source: Health Qual Life Outcomes. 2026 Mar 19;24:55. doi: 10.1186/s12955-026-02518-8 (PMC13123141; doi:10.1186/s12955-026-02518-8)
Supplement: Supplementary file 1 — Supplementary Material 1 [file 12955_2026_2518_MOESM1_ESM.docx]

**Supplementary Material**

**Health-Related Quality of Life in Immunocompromised Adults with Mild–Moderate COVID-19 Treated with Nirmatrelvir-Ritonavir: Results from the Randomized, Double-blinded EPIC-IC Trial**

Ruth Mokgokong^1^, Paul Cislo^2^, Elena Tudone^3^, Edward Weinstein^2^, Joseph C. Cappelleri^4^

^1^ Pfizer Ltd, Walton Oaks, Tadworth, KT20 7NS, UK | ^2^ Pfizer Inc, New York, New York, US | ^3^ Pfizer s.r.l., Milan, Italy | ^4^ Pfizer Inc, Groton, Connecticut, US

Corresponding author: Ruth Mokgokong | Email: [Ruth.Mokgokong@pfizer.com](mailto:Ruth.mokgokong@pfizer.com)

**Table of Contents**

[Supplementary methods 4](#_Toc213851751)

[US CDC criteria for moderate or severe immunocompromise 4](#_Toc213851752)

[SF-36 acute form 4](#_Toc213851753)

[EQ-5D-5L 4](#_Toc213851754)

[Statistical analysis 5](#_Toc213851755)

[Supplementary results 5](#_Toc213851756)

[SF-36 completion 5](#_Toc213851757)

[SF-36 domain scores 6](#_Toc213851758)

[EQ-5D-5L completion 6](#_Toc213851759)

[Supplementary tables 7](#_Toc213851760)

[Supplementary Table 1. SF-36 completion rates 7](#_Toc213851761)

[Supplementary Table 2. SF-36 Physical Functioning scores – overall sample (N=150) 8](#_Toc213851762)

[Supplementary Table 3. SF-36 Role-Physical scores – overall sample (N=150) 10](#_Toc213851763)

[Supplementary Table 4. SF-36 Bodily Pain scores – overall sample (N=150) 12](#_Toc213851764)

[Supplementary Table 5. SF-36 General Health scores – overall sample (N=150) 14](#_Toc213851765)

[Supplementary Table 6. SF-36 Vitality scores – overall sample (N=150) 16](#_Toc213851766)

[Supplementary Table 7. SF-36 Social Functioning scores – overall sample (N=150) 19](#_Toc213851767)

[Supplementary Table 8. SF-36 Role-Emotional scores – overall sample (N=150) 22](#_Toc213851768)

[Supplementary Table 9. SF-36 Mental Health scores – overall sample (N=150) 24](#_Toc213851769)

[Supplementary Table 10. SF-36 PCS scores – overall sample (N=150) 27](#_Toc213851770)

[Supplementary Table 11. SF-36 MCS scores – overall sample (N=150) 29](#_Toc213851771)

[Supplementary Table 12. EQ-5D-5L completion rates 31](#_Toc213851772)

[Supplementary Table 13. EQ-5D-5L Self-Care scores – overall sample (N=150) 32](#_Toc213851773)

[Supplementary Table 14. EQ-5D-5L Mobility scores – overall sample (N=150) 34](#_Toc213851774)

[Supplementary Table 15. EQ-5D-5L Usual Activity scores – overall sample (N=150) 36](#_Toc213851775)

[Supplementary Table 16. EQ-5D-5L Pain/Discomfort scores – overall sample (N=150) 38](#_Toc213851776)

[Supplementary Table 17. EQ-5D-5L Anxiety/Depression scores – overall sample (N=150) 40](#_Toc213851777)

[Supplementary Table 18. EQ-5D-5L Index scores 42](#_Toc213851778)

[Supplementary figures 44](#_Toc213851779)

[Supplementary Figure 1. SF-36 domain scores by treatment arm 46](#_Toc213851780)

[Supplementary Figure 2. SF-36 domain scores by IC severity and treatment arm 48](#_Toc213851781)

[Supplementary Figure 3. SF-36 Physical Component Summary scores by treatment arm in the overall sample 49](#_Toc213851782)

[Supplementary Figure 4. SF-36 Physical Component Summary scores by treatment arm in severe IC and non-severe IC subpopulations 51](#_Toc213851783)

[Supplementary Figure 5. SF-36 Mental Component Summary scores by treatment arm in the overall sample 52](#_Toc213851784)

[Supplementary Figure 6. SF-36 Mental Component Summary scores by treatment arm in severe IC and non-severe IC subpopulations 53](#_Toc213851785)

[Supplementary Figure 7. Participants reporting ‘no problems/none’ in EQ-5D-5L dimensions across treatment arms and by treatment arm 55](#_Toc213851786)

[Supplementary Figure 8. Participants reporting ‘no problems’/’none’ in EQ-5D-5L domain scores by IC severity and treatment arm 57](#_Toc213851787)

[Supplementary Figure 9. EQ-5D-5L Index scores by treatment arm in the overall sample 58](#_Toc213851788)

[Supplementary Figure 10. EQ-5D-5L Index scores by treatment arm in severe IC and non-severe IC subpopulations 59](#_Toc213851789)

[References 60](#_Toc213851790)

# Supplementary methods

### US CDC criteria for moderate or severe immunocompromise

Participants enrolling in EPIC-IC needed to meet ≥1 of the US Centers of Disease Control and Prevention criteria for moderate or severe immunocompromise: recipient of solid organ transplant and receiving immunosuppressive therapy; receipt of chimeric antigen receptor (CAR)-T-cell therapy or hematopoietic cell transplantation and either within 2 years of transplantation or receiving immunosuppressive therapy; moderate or severe primary immunodeficiency (e.g., DiGeorge syndrome); active or recent use of ≥1 immune-weakening medications (e.g., corticosteroids, chemotherapy, TNF blockers, anti-CD20 biologics); active immunosuppressive treatment for a solid tumor or hematological malignancy; or HIV infection with CD4 cell count <200 mm^3^ within 6 months before screening [1,2].

### SF-36 acute form

Participants rated the 36 items on a Likert-type scale (e.g., “How much bodily pain did you have during the past week? None, Very Mild, Mild, Moderate, Severe, Very severe.”).

Raw scores were determined for each health domain by averaging the scores for individual items in that domain. Each raw domain score was transformed to a 0–100 scale, with higher scores representing better health and well-being.

The PCS scores were primarily influenced by Physical Functioning, Role-Physical, Bodily Pain, and General Health subscales, and MCS scores were primarily influenced by Mental Health, Role-Emotional, Social Functioning, and Vitality subscales [3].

### EQ-5D-5L

Participants rated one item for each dimension using a five-point ordinal scale that ranges from “no problems” to “extreme problems”/“unable to” (e.g., “Pain/Discomfort: I have no pain or discomfort, I have slight pain or discomfort, I have moderate pain or discomfort, I have severe pain or discomfort, I have extreme pain or discomfort”) [4].

The van Hout value sets had been derived as a crosswalk from EQ-5D-3L value sets [5] and were selected for this study based on author judgment that they most closely approximated the value sets of the countries of the study population. First, the value set was used to derive coefficients for each level in each dimension. These coefficients were then applied to a participant’s responses to generate a summary index score, where a score of 1 represents the best possible health state (i.e., with no problems), 0 represents a health state equivalent to being dead, and scores less than 0 represent health states considered worse than being dead.

### Statistical analysis

In the mixed-effects longitudinal models, treatment, time, and treatment-by-time interaction were included as fixed effects, baseline (yes/no) was included as a covariate, and intercept and time were considered as random effects particular to each participant. The unstructured covariance structure was used to define covariance between random effects. Following the mixed-effects repeated measures approach, missing data were assumed to be missing at random and were imputed implicitly using maximum likelihood estimation [6]. That is, the model used observed patterns from available data points across the entire sample to account for missing observations without explicitly imputing missing values [6].

# Supplementary results

### SF-36 completion

All returned questionnaires were complete (i.e., all questions were answered). The baseline completion rate was numerically lower in the 15-day arm (38%) than in the 5-day arm (58%) or 10-day arm (56%). Post-baseline completion rates ranged from 83–90% across timepoints and were numerically higher in the 10-day arm (88%–96% across timepoints) than in the 5-day arm (75%–89%) or the 15-day arm (82%–88%).

Baseline completion was greater in the severe IC subpopulation (58%) than in the non-severe IC subpopulation (46%), whereas post-baseline completion rates were lower in the severe IC subpopulation (68%–84% across timepoints) than in the non-severe IC subpopulation (89%–94% across timepoints).

### SF-36 domain scores

In the severe IC subpopulation, mean baseline Physical Functioning and Role-Physical scores were numerically lower in the 5-day arm than in the 10-day arm or 15-day arm (Physical Functioning: 32.3 vs. 40.6 and 37.2; Role-Physical: 30.9 vs. 36.0 and 36.1) and mean baseline Mental Health scores were higher (47.7 vs. 43.0 and 42.5) (Supplementary Figure 1). Baseline Social Functioning scores varied across treatment arms in both subpopulations (Supplementary Figure 1).

Several SF-36 scores were not significantly increased from baseline in the severe IC subpopulation (Figure 3): General Health scores at Day 21 and Day 44, Vitality scores at Day 10 and Day 44, Social Functioning scores at Day 10, and Mental Health scores at Day 44.

### EQ-5D-5L completion

All returned questionnaires were complete. Both baseline and post-baseline EQ-5D-5L completion rates were generally similar across treatment arms, except that the Week 24 completion rate was numerically lower in the 5-day arm (75%) than in the 10-day arm (90%) or 15-day arm (84%) (Supplementary Table 12).

Post-baseline completion rates were lower in the severe IC subpopulation (67–82% across timepoints) than in the non-severe IC subpopulation (90–94% across timepoints). All returned questionnaires were complete.

## Supplementary tables

### Supplementary Table 1. SF-36 completion rates

| **Time Point** | **5-day NMV/r** | **10-day NMV/r** | **15-day NMV/r** | **All arms** |
| --- | --- | --- | --- | --- |
| ***Overall sample*** | ***N=52*** | ***N=48*** | ***N=50*** | ***N=150*** |
| Baseline (Day 1) | 30 (57.7%) | 27 (56.3%) | 19 (38.0%) | 76 (50.7%) |
| Day 10 | 46 (88.5%) | 46 (95.8%) | 43 (86.0%) | 135 (90.0%) |
| Day 21 | 42 (80.8%) | 42 (87.5%) | 43 (86.0%) | 127 (84.7%) |
| Day 44 | 43 (82.7%) | 44 (91.7%) | 44 (88.0%) | 131 (87.3%) |
| Week 12 | 44 (84.6%) | 42 (87.5%) | 41 (82.0%) | 127 (84.7%) |
| Week 24 | 39 (75.0%) | 44 (91.7%) | 42 (84.0%) | 125 (83.3%) |
| ***Severe IC*** | ***N=20*** | ***N=17*** | ***N=20*** | ***N=57*** |
| Baseline (Day 1) | 12 (60.0%) | 12 (70.6%) | 9 (45.0%) | 33 (57.9%) |
| Day 10 | 17 (85.0%) | 16 (94.1%) | 15 (75.0%) | 48 (84.2%) |
| Day 21 | 15 (75.0%) | 15 (88.2%) | 14 (70.0%) | 44 (77.2%) |
| Day 44 | 15 (75.0%) | 16 (94.1%) | 15 (75.0%) | 46 (80.7%) |
| Week 12 | 13 (65.0%) | 13 (76.5%) | 13 (65.0%) | 39 (68.4%) |
| Week 24 | 13 (65.0%) | 15 (88.2%) | 13 (65.0%) | 41 (71.9%) |
| ***Non-severe IC*** | ***N=32*** | ***N=31*** | ***N=30*** | ***N=93*** |
| Baseline (Day 1) | 18 (56.3%) | 15 (48.4%) | 10 (33.3%) | 43 (46.2%) |
| Day 10 | 29 (90.6%) | 30 (96.8%) | 28 (93.3%) | 87 (93.5%) |
| Day 21 | 27 (84.4%) | 27 (87.1%) | 29 (96.7%) | 83 (89.2%) |
| Day 44 | 28 (87.5%) | 28 (90.3%) | 29 (96.7%) | 85 (91.4%) |
| Week 12 | 31 (96.9%) | 29 (93.5%) | 28 (93.3%) | 88 (94.6%) |
| Week 24 | 26 (81.3%) | 29 (93.5%) | 29 (96.7%) | 84 (90.3%) |

Abbreviations: IC, immunocompromise; NMV/r, nirmatrelvir-ritonavir; SF-36, 36-Item Short Form Health Survey acute form.

### Supplementary Table 2. SF-36 Physical Functioning scores – overall sample (N=150)

| Visit | 5-day NMV/r (N=52) | 10-day NMV/r (N=48) | 15-day NMV/r (N=50) | All arms (N=150) |
| --- | --- | --- | --- | --- |
| **Baseline (Day 1)** |  |  |  |  |
| *Observed* |  |  |  |  |
| n | 48 | 46 | 46 | 140 |
| Mean (SD) | 35.410 (10.744) | 39.138 (11.439) | 38.738 (10.067) | 37.728 (10.819) |
| SE | 1.551 | 1.687 | 1.484 | 0.914 |
| Median (range) | 36.647 (18.228, 57.112) | 39.716 (16.181, 57.112) | 37.670 (18.228, 57.112) | 36.647 (16.181, 57.112) |
| 95% CI | [32.290, 38.530] | [35.741, 42.535] | [35.748, 41.727] | [35.920, 39.536] |
|  |  |  |  |  |
| **Day 10** |  |  |  |  |
| *Observed* |  |  |  |  |
| n | 46 | 46 | 43 | 135 |
| Mean (SD) | 42.252 (10.598) | 47.413 (8.899) | 45.213 (10.561) | 44.954 (10.192) |
| SE | 1.563 | 1.312 | 1.611 | 0.877 |
| Median (range) | 44.833 (20.275, 57.112) | 48.926 (28.461, 57.112) | 46.879 (20.275, 57.112) | 46.879 (20.275, 57.112) |
| 95% CI | [39.105, 45.400] | [44.770, 50.056] | [41.963, 48.464] | [43.219, 46.689] |
| *Change from baseline* |  |  |  |  |
| n | 44 | 45 | 39 | 128 |
| Mean (SD) | 6.698 (10.078) | 8.641 (11.217) | 6.244 (7.911) | 7.243 (9.890) |
| SE | 1.519 | 1.672 | 1.267 | 0.874 |
| Median (range) | 5.116 (-20.465, 32.744) | 6.140 (-8.186, 40.930) | 6.140 (-12.279, 22.512) | 6.140 (-20.465, 40.930) |
| 95% CI | [3.634, 9.762] | [5.271, 12.011] | [3.680, 8.809] | [5.513, 8.973] |
|  |  |  |  |  |
| **Day 21** |  |  |  |  |
| *Observed* |  |  |  |  |
| n | 42 | 42 | 43 | 127 |
| Mean (SD) | 44.638 (11.217) | 49.072 (9.719) | 48.307 (7.933) | 47.347 (9.819) |
| SE | 1.731 | 1.500 | 1.210 | 0.871 |
| Median (range) | 46.879 (18.228, 57.112) | 53.019 (22.321, 57.112) | 50.972 (28.461, 57.112) | 50.972 (18.228, 57.112) |
| 95% CI | [41.142, 48.133] | [46.043, 52.101] | [45.866, 50.748] | [45.622, 49.071] |
| *Change from baseline* |  |  |  |  |
| n | 40 | 41 | 40 | 121 |
| Mean (SD) | 8.749 (9.407) | 9.534 (11.326) | 10.181 (8.284) | 9.488 (9.704) |
| SE | 1.487 | 1.769 | 1.310 | 0.882 |
| Median (range) | 8.186 (-16.372, 28.651) | 8.186 (-6.140, 40.930) | 8.186 (-6.140, 32.744) | 8.186 (-16.372, 40.930) |
| 95% CI | [5.740, 11.757] | [5.959, 13.109] | [7.532, 12.831] | [7.742, 11.235] |
|  |  |  |  |  |
| **Day 44** |  |  |  |  |
| *Observed* |  |  |  |  |
| n | 43 | 44 | 44 | 131 |
| Mean (SD) | 47.165 (10.742) | 47.577 (11.660) | 48.182 (8.258) | 47.645 (10.246) |
| SE | 1.638 | 1.758 | 1.245 | 0.895 |
| Median (range) | 50.972 (20.275, 57.112) | 51.996 (18.228, 57.112) | 48.926 (26.414, 57.112) | 50.972 (18.228, 57.112) |
| 95% CI | [43.859, 50.471] | [44.032, 51.122] | [45.671, 50.692] | [45.874, 49.416] |
| *Change from baseline* |  |  |  |  |
| n | 41 | 43 | 41 | 125 |
| Mean (SD) | 10.582 (9.231) | 8.281 (13.111) | 9.883 (9.197) | 9.561 (10.675) |
| SE | 1.442 | 1.999 | 1.436 | 0.955 |
| Median (range) | 10.233 (-2.047, 30.698) | 8.186 (-32.744, 40.930) | 10.233 (-8.186, 34.791) | 8.186 (-32.744, 40.930) |
| 95% CI | [7.668, 13.496] | [4.246, 12.316] | [6.980, 12.786] | [7.672, 11.451] |
|  |  |  |  |  |
| **Week 12** |  |  |  |  |
| *Observed* |  |  |  |  |
| n | 44 | 42 | 41 | 127 |
| Mean (SD) | 49.158 (9.260) | 49.023 (9.185) | 48.676 (9.579) | 48.958 (9.268) |
| SE | 1.396 | 1.417 | 1.496 | 0.822 |
| Median (range) | 51.996 (20.275, 57.112) | 53.019 (26.414, 57.112) | 50.972 (18.228, 57.112) | 50.972 (18.228, 57.112) |
| 95% CI | [46.343, 51.974] | [46.161, 51.886] | [45.653, 51.700] | [47.331, 50.585] |
| *Change from baseline* |  |  |  |  |
| n | 42 | 41 | 39 | 122 |
| Mean (SD) | 12.718 (11.214) | 10.981 (11.104) | 10.442 (10.155) | 11.407 (10.803) |
| SE | 1.730 | 1.734 | 1.626 | 0.978 |
| Median (range) | 11.256 (-6.140, 38.884) | 8.186 (-10.233, 40.930) | 10.233 (-14.326, 34.791) | 10.233 (-14.326, 40.930) |
| 95% CI | [9.223, 16.212] | [7.477, 14.486] | [7.151, 13.734] | [9.470, 13.343] |
|  |  |  |  |  |
| *Week 24* |  |  |  |  |
| **Observed** |  |  |  |  |
| n | 39 | 44 | 42 | 125 |
| Mean (SD) | 47.719 (12.032) | 48.693 (10.741) | 48.926 (9.684) | 48.467 (10.754) |
| SE | 1.927 | 1.619 | 1.494 | 0.962 |
| Median (range) | 55.065 (18.228, 57.112) | 53.019 (18.228, 57.112) | 50.972 (24.368, 57.112) | 53.019 (18.228, 57.112) |
| 95% CI | [43.819, 51.619] | [45.428, 51.959] | [45.908, 51.943] | [46.564, 50.371] |
| *Change from baseline* |  |  |  |  |
| n | 37 | 43 | 39 | 119 |
| Mean (SD) | 11.892 (14.144) | 10.280 (11.538) | 10.338 (12.641) | 10.800 (12.666) |
| SE | 2.325 | 1.760 | 2.024 | 1.161 |
| Median (range) | 10.233 (-16.372, 38.884) | 6.140 (-10.233, 40.930) | 10.233 (-18.419, 34.791) | 8.186 (-18.419, 40.930) |
| 95% CI | [7.176, 16.608] | [6.729, 13.831] | [6.240, 14.435] | [8.501, 13.099] |

Abbreviations: CI, confidence interval; PF, Physical Functioning; NMV/r, nirmatrelvir-ritonavir; SD, standard deviation; SE, standard error; SF-36, 36-Item Short Form Health Survey.

### Supplementary Table 3. SF-36 Role-Physical scores – overall sample (N=150)

| Visit | 5-day NMV/r (N=52) | 10-day NMV/r (N=48) | 15-day NMV/r (N=50) | All arms (N=150) |
| --- | --- | --- | --- | --- |
| **Baseline (Day 1)** |  |  |  |  |
| *Observed* |  |  |  |  |
| n | 48 | 46 | 46 | 140 |
| Mean (SD) | 35.546 (10.988) | 34.733 (10.002) | 36.912 (9.863) | 35.728 (10.272) |
| SE | 1.586 | 1.475 | 1.454 | 0.868 |
| Median (range) | 37.534 (18.445, 56.624) | 36.341 (18.445, 54.237) | 36.341 (18.445, 56.624) | 37.534 (18.445, 56.624) |
| 95% CI | [32.355, 38.736] | [31.763, 37.703] | [33.983, 39.841] | [34.011, 37.444] |
|  |  |  |  |  |
| **Day 10** |  |  |  |  |
| *Observed* |  |  |  |  |
| n | 46 | 46 | 43 | 135 |
| Mean (SD) | 38.053 (10.442) | 41.373 (8.749) | 40.531 (10.071) | 39.974 (9.806) |
| SE | 1.540 | 1.290 | 1.536 | 0.844 |
| Median (range) | 37.534 (18.445, 56.624) | 39.920 (25.604, 56.624) | 37.534 (18.445, 56.624) | 37.534 (18.445, 56.624) |
| 95% CI | [34.952, 41.154] | [38.775, 43.971] | [37.431, 43.630] | [38.304, 41.643] |
| *Change from baseline* |  |  |  |  |
| n | 44 | 45 | 39 | 128 |
| Mean (SD) | 2.332 (10.971) | 6.522 (10.306) | 2.998 (9.204) | 4.008 (10.316) |
| SE | 1.654 | 1.536 | 1.474 | 0.912 |
| Median (range) | 0.000 (-28.634, 21.475) | 4.772 (-21.475, 28.634) | 2.386 (-14.317, 28.634) | 2.386 (-28.634, 28.634) |
| 95% CI | [-1.004, 5.667] | [3.426, 9.618] | [0.014, 5.982] | [2.204, 5.812] |
|  |  |  |  |  |
| **Day 21** |  |  |  |  |
| *Observed* |  |  |  |  |
| n | 42 | 42 | 43 | 127 |
| Mean (SD) | 44.295 (10.054) | 47.533 (10.222) | 47.689 (8.869) | 46.515 (9.776) |
| SE | 1.551 | 1.577 | 1.352 | 0.867 |
| Median (range) | 47.079 (18.445, 56.624) | 49.465 (18.445, 56.624) | 47.079 (27.990, 56.624) | 47.079 (18.445, 56.624) |
| 95% CI | [41.162, 47.428] | [44.348, 50.719] | [44.960, 50.419] | [44.799, 48.232] |
| *Change from baseline* |  |  |  |  |
| n | 40 | 41 | 40 | 121 |
| Mean (SD) | 7.874 (11.617) | 11.756 (12.876) | 10.559 (10.265) | 10.077 (11.663) |
| SE | 1.837 | 2.011 | 1.623 | 1.060 |
| Median (range) | 8.352 (-14.317, 28.634) | 9.545 (-19.089, 38.178) | 9.545 (-16.703, 33.406) | 9.545 (-19.089, 38.178) |
| 95% CI | [4.159, 11.590] | [7.692, 15.820] | [7.276, 13.842] | [7.978, 12.176] |
|  |  |  |  |  |
| **Day 44** |  |  |  |  |
| *Observed* |  |  |  |  |
| n | 43 | 44 | 44 | 131 |
| Mean (SD) | 45.747 (11.037) | 46.049 (12.007) | 48.272 (8.432) | 46.696 (10.576) |
| SE | 1.683 | 1.810 | 1.271 | 0.924 |
| Median (range) | 47.079 (18.445, 56.624) | 49.465 (18.445, 56.624) | 47.079 (27.990, 56.624) | 49.465 (18.445, 56.624) |
| 95% CI | [42.350, 49.144] | [42.398, 49.699] | [45.708, 50.836] | [44.868, 48.524] |
| *Change from baseline* | 41 | 43 | 41 | 125 |
| n | 9.777 (12.925) | 10.765 (14.546) | 10.941 (10.165) | 10.499 (12.615) |
| Mean (SD) | 43 | 44 | 44 | 131 |
| SE | 2.019 | 2.218 | 1.588 | 1.128 |
| Median (range) | 9.545 (-9.545, 35.792) | 9.545 (-21.475, 38.178) | 9.545 (-9.545, 33.406) | 9.545 (-21.475, 38.178) |
| 95% CI | [5.698, 13.857] | [6.289, 15.242] | [7.733, 14.150] | [8.266, 12.732] |
|  |  |  |  |  |
| **Week 12** |  |  |  |  |
| *Observed* |  |  |  |  |
| n | 44 | 42 | 41 | 127 |
| Mean (SD) | 49.140 (9.314) | 47.761 (9.835) | 47.836 (8.477) | 48.263 (9.182) |
| SE | 1.404 | 1.518 | 1.324 | 0.815 |
| Median (range) | 53.044 (20.831, 56.624) | 49.465 (20.831, 56.624) | 47.079 (27.990, 56.624) | 49.465 (20.831, 56.624) |
| 95% CI | [46.308, 51.971] | [44.696, 50.826] | [45.160, 50.511] | [46.650, 49.875] |
| *Change from baseline* |  |  |  |  |
| n | 42 | 41 | 39 | 122 |
| Mean (SD) | 11.988 (13.585) | 13.386 (13.433) | 11.074 (9.922) | 12.165 (12.410) |
| SE | 2.096 | 2.098 | 1.589 | 1.124 |
| Median (range) | 9.545 (-11.931, 38.178) | 14.317 (-11.931, 38.178) | 9.545 (-11.931, 33.406) | 9.545 (-11.931, 38.178) |
| 95% CI | [7.754, 16.221] | [9.146, 17.626] | [7.858, 14.290] | [9.941, 14.390] |
|  |  |  |  |  |
| **Week 24** |  |  |  |  |
| *Observed* |  |  |  |  |
| n | 39 | 44 | 42 | 125 |
| Mean (SD) | 47.018 (11.528) | 48.326 (10.401) | 47.249 (11.381) | 47.556 (11.018) |
| SE | 1.846 | 1.568 | 1.756 | 0.986 |
| Median (range) | 49.465 (18.445, 56.624) | 51.851 (20.831, 56.624) | 53.044 (18.445, 56.624) | 51.851 (18.445, 56.624) |
| 95% CI | [43.281, 50.755] | [45.164, 51.488] | [43.703, 50.796] | [45.606, 49.507] |
| *Change from baseline* |  |  |  |  |
| n | 37 | 43 | 39 | 119 |
| Mean (SD) | 10.125 (14.595) | 13.706 (13.689) | 9.789 (12.445) | 11.309 (13.596) |
| SE | 2.399 | 2.087 | 1.993 | 1.246 |
| Median (range) | 7.158 (-21.475, 38.178) | 14.317 (-16.703, 38.178) | 9.545 (-19.089, 33.406) | 9.545 (-21.475, 38.178) |
| 95% CI | [5.259, 14.991] | [9.494, 17.919] | [5.755, 13.823] | [8.841, 13.777] |

Abbreviations: CI, confidence interval; NMV/r, nirmatrelvir-ritonavir; SD, standard deviation; SE, standard error; SF-36, 36-Item Short Form Health Survey.

### Supplementary Table 4. SF-36 Bodily Pain scores – overall sample (N=150)

| Visit | 5-day NMV/r (N=52) | 10-day NMV/r (N=48) | 15-day NMV/r (N=50) | All arms (N=150) |
| --- | --- | --- | --- | --- |
| **Baseline (Day 1)** |  |  |  |  |
| *Observed* |  |  |  |  |
| n | 48 | 46 | 46 | 140 |
| Mean (SD) | 42.227 (10.208) | 39.732 (9.461) | 41.579 (10.719) | 41.194 (10.126) |
| SE | 1.473 | 1.395 | 1.580 | 0.856 |
| Median (range) | 40.683 (23.397, 60.883) | 40.683 (19.232, 60.883) | 40.683 (19.232, 60.883) | 40.683 (19.232, 60.883) |
| 95% CI | [39.263, 45.191] | [36.922, 42.541] | [38.396, 44.762] | [39.502, 42.886] |
|  |  |  |  |  |
| **Day 10** |  |  |  |  |
| *Observed* |  |  |  |  |
| n | 46 | 46 | 43 | 135 |
| Mean (SD) | 48.470 (10.015) | 48.479 (9.487) | 48.194 (10.820) | 48.385 (10.031) |
| SE | 1.477 | 1.399 | 1.650 | 0.863 |
| Median (range) | 50.054 (19.232, 60.883) | 50.054 (28.395, 60.883) | 50.054 (23.397, 60.883) | 50.054 (19.232, 60.883) |
| 95% CI | [45.495, 51.444] | [45.661, 51.296] | [44.864, 51.524] | [46.677, 50.093] |
| *Change from baseline* |  |  |  |  |
| n | 44 | 45 | 39 | 128 |
| Mean (SD) | 6.333 (11.874) | 8.580 (9.050) | 5.457 (10.883) | 6.856 (10.644) |
| SE | 1.790 | 1.349 | 1.743 | 0.941 |
| Median (range) | 7.705 (-19.993, 32.905) | 7.914 (-9.580, 24.574) | 4.165 (-19.993, 28.739) | 6.664 (-19.993, 32.905) |
| 95% CI | [2.723, 9.943] | [5.861, 11.299] | [1.930, 8.985] | [4.994, 8.718] |
|  |  |  |  |  |
| **Day 21** |  |  |  |  |
| *Observed* |  |  |  |  |
| n | 42 | 42 | 43 | 127 |
| Mean (SD) | 50.153 (9.843) | 52.018 (10.500) | 51.633 (8.859) | 51.271 (9.706) |
| SE | 1.519 | 1.620 | 1.351 | 0.861 |
| Median (range) | 50.054 (19.232, 60.883) | 57.551 (28.395, 60.883) | 52.553 (32.144, 60.883) | 52.553 (19.232, 60.883) |
| 95% CI | [47.086, 53.221] | [48.746, 55.290] | [48.907, 54.359] | [49.566, 52.975] |
| *Change from baseline* |  |  |  |  |
| n | 40 | 41 | 40 | 121 |
| Mean (SD) | 7.549 (9.557) | 11.398 (10.320) | 10.309 (12.074) | 9.766 (10.734) |
| SE | 1.511 | 1.612 | 1.909 | 0.976 |
| Median (range) | 8.747 (-19.993, 24.574) | 10.829 (-9.163, 32.488) | 10.205 (-19.993, 36.653) | 9.163 (-19.993, 36.653) |
| 95% CI | [4.493, 10.606] | [8.141, 14.656] | [6.447, 14.170] | [7.834, 11.698] |
|  |  |  |  |  |
| **Day 44** |  |  |  |  |
| *Observed* |  |  |  |  |
| n | 43 | 44 | 44 | 131 |
| Mean (SD) | 53.803 (9.067) | 50.811 (11.701) | 51.067 (11.111) | 51.879 (10.701) |
| SE | 1.383 | 1.764 | 1.675 | 0.935 |
| Median (range) | 60.883 (32.144, 60.883) | 54.219 (23.397, 60.883) | 54.219 (28.395, 60.883) | 54.219 (23.397, 60.883) |
| 95% CI | [51.012, 56.593] | [47.254, 54.369] | [47.689, 54.445] | [50.029, 53.729] |
| *Change from baseline* |  |  |  |  |
| n | 41 | 43 | 41 | 125 |
| Mean (SD) | 11.429 (13.317) | 10.616 (13.808) | 9.478 (12.181) | 10.509 (13.049) |
| SE | 2.080 | 2.106 | 1.902 | 1.167 |
| Median (range) | 12.912 (-17.910, 32.905) | 9.163 (-16.244, 41.651) | 8.747 (-16.661, 32.488) | 10.829 (-17.910, 41.651) |
| 95% CI | [7.225, 15.632] | [6.367, 14.866] | [5.633, 13.323] | [8.199, 12.820] |
|  |  |  |  |  |
| **Week 12** |  |  |  |  |
| *Observed* |  |  |  |  |
| n | 44 | 42 | 41 | 127 |
| Mean (SD) | 55.033 (9.268) | 50.848 (11.216) | 51.456 (11.276) | 52.494 (10.683) |
| SE | 1.397 | 1.731 | 1.761 | 0.948 |
| Median (range) | 60.883 (32.144, 60.883) | 54.219 (24.230, 60.883) | 54.219 (28.395, 60.883) | 60.883 (24.230, 60.883) |
| 95% CI | [52.216, 57.851] | [47.352, 54.343] | [47.897, 55.015] | [50.618, 54.370] |
| *Change from baseline* |  |  |  |  |
| n | 42 | 41 | 39 | 122 |
| Mean (SD) | 12.247 (13.941) | 11.358 (12.133) | 10.242 (11.273) | 11.307 (12.454) |
| SE | 2.151 | 1.895 | 1.805 | 1.128 |
| Median (range) | 14.578 (-28.739, 32.905) | 13.328 (-17.910, 32.488) | 9.163 (-12.495, 36.653) | 11.662 (-28.739, 36.653) |
| 95% CI | [7.903, 16.592] | [7.528, 15.187] | [6.588, 13.896] | [9.075, 13.540] |
|  |  |  |  |  |
| **Week 24** |  |  |  |  |
| *Observed* |  |  |  |  |
| n | 39 | 44 | 42 | 125 |
| Mean (SD) | 52.585 (11.522) | 51.834 (10.592) | 50.719 (11.064) | 51.694 (10.984) |
| SE | 1.845 | 1.597 | 1.707 | 0.982 |
| Median (range) | 60.883 (19.232, 60.883) | 54.219 (24.230, 60.883) | 53.386 (24.230, 60.883) | 54.219 (19.232, 60.883) |
| 95% CI | [48.850, 56.320] | [48.613, 55.054] | [47.271, 54.166] | [49.749, 53.638] |
| *Change from baseline* |  |  |  |  |
| n | 37 | 43 | 39 | 119 |
| Mean (SD) | 10.199 (15.683) | 12.108 (11.364) | 8.672 (12.656) | 10.388 (13.215) |
| SE | 2.578 | 1.733 | 2.027 | 1.211 |
| Median (range) | 11.662 (-28.739, 36.653) | 11.662 (-8.330, 36.653) | 4.998 (-20.409, 36.653) | 10.829 (-28.739, 36.653) |
| 95% CI | [4.970, 15.428] | [8.611, 15.605] | [4.569, 12.775] | [7.989, 12.787] |

Abbreviations: CI, confidence interval; NMV/r, nirmatrelvir-ritonavir; SD, standard deviation; SE, standard error; SF-36, 36-Item Short Form Health Survey.

### Supplementary Table 5. SF-36 General Health scores – overall sample (N=150)

|  | 5-day NMV/r (N=52) | 10-day NMV/r (N=48) | 15-day NMV/r (N=50) | All arms (N=150) |
| --- | --- | --- | --- | --- |
| **Baseline (Day 1)** |  |  |  |  |
| *Observed* |  |  |  |  |
| n | 48 | 46 | 46 | 140 |
| Mean (SD) | 40.521 (9.273) | 38.226 (10.407) | 38.767 (10.798) | 39.191 (10.142) |
| SE | 1.338 | 1.534 | 1.592 | 0.857 |
| Median (range) | 39.533 (23.797, 63.724) | 37.184 (21.448, 61.375) | 37.889 (21.448, 61.375) | 37.889 (21.448, 63.724) |
| 95% CI | [37.829, 43.214] | [35.135, 41.316] | [35.560, 41.974] | [37.496, 40.885] |
|  |  |  |  |  |
| **Day 10** |  |  |  |  |
| *Observed* |  |  |  |  |
| n | 46 | 46 | 43 | 135 |
| Mean (SD) | 42.749 (9.102) | 42.218 (10.388) | 41.985 (12.099) | 42.325 (10.490) |
| SE | 1.342 | 1.532 | 1.845 | 0.903 |
| Median (range) | 41.177 (26.146, 63.724) | 41.177 (21.448, 62.314) | 42.586 (21.448, 63.724) | 41.177 (21.448, 63.724) |
| 95% CI | [40.046, 45.452] | [39.133, 45.303] | [38.262, 45.709] | [40.539, 44.111] |
| *Change from baseline* |  |  |  |  |
| n | 44 | 45 | 39 | 128 |
| Mean (SD) | 1.623 (7.343) | 4.134 (7.501) | 2.854 (7.807) | 2.881 (7.556) |
| SE | 1.107 | 1.118 | 1.250 | 0.668 |
| Median (range) | 2.349 (-18.789, 16.440) | 4.697 (-11.743, 24.426) | 2.349 (-22.077, 26.774) | 2.349 (-22.077, 26.774) |
| 95% CI | [-0.610, 3.855] | [1.880, 6.387] | [0.324, 5.385] | [1.559, 4.202] |
|  |  |  |  |  |
| **Day 21** |  |  |  |  |
| *Observed* |  |  |  |  |
| n | 42 | 42 | 43 | 127 |
| Mean (SD) | 42.385 (10.143) | 43.548 (11.612) | 42.662 (11.949) | 42.863 (11.189) |
| SE | 1.565 | 1.792 | 1.822 | 0.993 |
| Median (range) | 41.177 (23.797, 63.724) | 41.177 (19.100, 63.724) | 41.177 (19.100, 63.724) | 41.177 (19.100, 63.724) |
| 95% CI | [39.224, 45.545] | [39.929, 47.166] | [38.985, 46.340] | [40.899, 44.828] |
| *Change from baseline* |  |  |  |  |
| n | 40 | 41 | 40 | 121 |
| Mean (SD) | 1.280 (8.372) | 5.385 (9.114) | 3.687 (7.280) | 3.467 (8.400) |
| SE | 1.324 | 1.423 | 1.151 | 0.764 |
| Median (range) | 2.349 (-18.789, 24.895) | 3.288 (-11.743, 35.229) | 3.288 (-17.380, 25.835) | 2.349 (-18.789, 35.229) |
| 95% CI | [-1.398, 3.958] | [2.508, 8.261] | [1.359, 6.016] | [1.955, 4.979] |
|  |  |  |  |  |
| **Day 44** |  |  |  |  |
| *Observed* |  |  |  |  |
| n | 43 | 44 | 44 | 131 |
| Mean (SD) | 44.552 (10.684) | 41.988 (12.911) | 41.945 (11.510) | 42.815 (11.718) |
| SE | 1.629 | 1.946 | 1.735 | 1.024 |
| Median (range) | 42.586 (26.146, 63.724) | 40.002 (16.751, 63.724) | 41.177 (21.448, 63.724) | 41.177 (16.751, 63.724) |
| 95% CI | [41.264, 47.840] | [38.063, 45.913] | [38.446, 45.445] | [40.790, 44.841] |
| *Change from baseline* |  |  |  |  |
| n | 41 | 43 | 41 | 125 |
| Mean (SD) | 3.368 (8.471) | 4.107 (10.456) | 2.807 (8.297) | 3.438 (9.092) |
| SE | 1.323 | 1.594 | 1.296 | 0.813 |
| Median (range) | 3.288 (-11.743, 24.895) | 2.349 (-18.789, 35.229) | 4.697 (-18.789, 24.426) | 3.288 (-18.789, 35.229) |
| 95% CI | [0.695, 6.042] | [0.890, 7.325] | [0.188, 5.426] | [1.829, 5.048] |
|  |  |  |  |  |
| **Week 12** |  |  |  |  |
| *Observed* |  |  |  |  |
| n | 44 | 42 | 41 | 127 |
| Mean (SD) | 45.394 (9.295) | 43.380 (12.312) | 43.102 (12.257) | 43.988 (11.295) |
| SE | 1.401 | 1.900 | 1.914 | 1.002 |
| Median (range) | 43.525 (26.146, 63.724) | 41.177 (21.448, 63.724) | 41.177 (21.448, 63.724) | 41.177 (21.448, 63.724) |
| 95% CI | [42.568, 48.220] | [39.543, 47.217] | [39.233, 46.970] | [42.004, 45.971] |
| *Change from baseline* |  |  |  |  |
| n | 42 | 41 | 39 | 122 |
| Mean (SD) | 3.825 (7.963) | 5.694 (10.324) | 4.228 (8.524) | 4.582 (8.956) |
| SE | 1.229 | 1.612 | 1.365 | 0.811 |
| Median (range) | 4.697 (-10.804, 24.895) | 3.758 (-18.789, 35.229) | 3.288 (-18.789, 29.123) | 4.228 (-18.789, 35.229) |
| 95% CI | [1.343, 6.306] | [2.435, 8.953] | [1.464, 6.991] | [2.977, 6.187] |
|  |  |  |  |  |
| **Week 24** |  |  |  |  |
| *Observed* |  |  |  |  |
| n | 39 | 44 | 42 | 125 |
| Mean (SD) | 45.091 (11.725) | 43.878 (12.290) | 44.018 (12.237) | 44.303 (12.013) |
| SE | 1.878 | 1.853 | 1.888 | 1.074 |
| Median (range) | 45.874 (16.751, 63.724) | 41.177 (21.448, 63.724) | 43.525 (19.100, 63.724) | 43.525 (16.751, 63.724) |
| 95% CI | [41.290, 48.892] | [40.141, 47.614] | [40.204, 47.831] | [42.177, 46.430] |
| *Change from baseline* |  |  |  |  |
| n | 37 | 43 | 39 | 119 |
| Mean (SD) | 4.202 (8.111) | 6.412 (10.023) | 4.517 (8.995) | 5.104 (9.104) |
| SE | 1.333 | 1.529 | 1.440 | 0.835 |
| Median (range) | 4.697 (-12.683, 24.895) | 4.697 (-11.743, 35.229) | 4.697 (-24.426, 26.774) | 4.697 (-24.426, 35.229) |
| 95% CI | [1.498, 6.906] | [3.328, 9.497] | [1.601, 7.432] | [3.451, 6.756] |

Abbreviations: CI, confidence interval; NMV/r, nirmatrelvir-ritonavir; SD, standard deviation; SE, standard error; SF-36, 36-Item Short Form Health Survey.

### Supplementary Table 6. SF-36 Vitality scores – overall sample (N=150)

| Visit | 5-day NMV/r (N=52) | 10-day NMV/r (N=48) | 15-day NMV/r (N=50) | All arms (N=150) |
| --- | --- | --- | --- | --- |
| **Baseline (Day 1)** |  |  |  |  |
| *Observed* |  |  |  |  |
| n | 48 | 46 | 46 | 140 |
| Mean (SD) | 41.851 (11.464) | 40.501 (10.566) | 43.169 (11.948) | 41.840 (11.312) |
| SE | 1.655 | 1.558 | 1.762 | 0.956 |
| Median (range) | 42.974 (22.019, 69.916) | 39.980 (22.019, 66.922) | 44.470 (22.019, 69.916) | 42.974 (22.019, 69.916) |
| 95% CI | [38.522, 45.180] | [37.363, 43.638] | [39.621, 46.717] | [39.950, 43.731] |
|  |  |  |  |  |
| **Day 10** |  |  |  |  |
| *Observed* |  |  |  |  |
| n | 46 | 46 | 43 | 135 |
| Mean (SD) | 44.601 (13.707) | 45.837 (11.498) | 47.986 (11.969) | 46.100 (12.423) |
| SE | 2.021 | 1.695 | 1.825 | 1.069 |
| Median (range) | 42.974 (22.019, 69.916) | 48.961 (22.019, 69.916) | 48.961 (22.019, 69.916) | 45.967 (22.019, 69.916) |
| 95% CI | [40.530, 48.671] | [42.423, 49.252] | [44.303, 51.670] | [43.986, 48.215] |
| *Change from baseline* |  |  |  |  |
| n | 44 | 45 | 39 | 128 |
| Mean (SD) | 2.585 (10.620) | 5.056 (9.633) | 3.531 (9.758) | 3.742 (9.996) |
| SE | 1.601 | 1.436 | 1.562 | 0.884 |
| Median (range) | 2.994 (-20.955, 29.935) | 5.987 (-20.955, 29.935) | 0.000 (-17.961, 26.942) | 2.994 (-20.955, 29.935) |
| 95% CI | [-0.643, 5.814] | [2.162, 7.950] | [0.368, 6.694] | [1.994, 5.490] |
|  |  |  |  |  |
| **Day 21** |  |  |  |  |
| *Observed* |  |  |  |  |
| n | 42 | 42 | 43 | 127 |
| Mean (SD) | 47.321 (12.909) | 52.168 (12.288) | 50.492 (11.016) | 49.998 (12.159) |
| SE | 1.992 | 1.896 | 1.680 | 1.079 |
| Median (range) | 45.967 (22.019, 69.916) | 53.451 (22.019, 69.916) | 48.961 (22.019, 69.916) | 48.961 (22.019, 69.916) |
| 95% CI | [43.299, 51.344] | [48.339, 55.997] | [47.102, 53.883] | [47.863, 52.133] |
| *Change from baseline* |  |  |  |  |
| n | 40 | 41 | 40 | 121 |
| Mean (SD) | 5.538 (12.710) | 11.171 (12.523) | 6.286 (8.834) | 7.694 (11.683) |
| SE | 2.010 | 1.956 | 1.397 | 1.062 |
| Median (range) | 5.987 (-23.948, 38.916) | 8.981 (-17.961, 35.923) | 5.987 (-14.968, 29.935) | 5.987 (-23.948, 38.916) |
| 95% CI | [1.473, 9.603] | [7.218, 15.124] | [3.461, 9.112] | [5.591, 9.797] |
|  |  |  |  |  |
| **Day 44** |  |  |  |  |
| *Observed* |  |  |  |  |
| n | 43 | 44 | 44 | 131 |
| Mean (SD) | 49.866 (12.108) | 50.049 (14.752) | 50.934 (11.584) | 50.286 (12.804) |
| SE | 1.846 | 2.224 | 1.746 | 1.119 |
| Median (range) | 48.961 (25.012, 69.916) | 51.954 (22.019, 69.916) | 51.954 (25.012, 69.916) | 51.954 (22.019, 69.916) |
| 95% CI | [46.140, 53.592] | [45.564, 54.534] | [47.412, 54.456] | [48.073, 52.499] |
| *Change from baseline* |  |  |  |  |
| n | 41 | 43 | 41 | 125 |
| Mean (SD) | 7.739 (13.253) | 9.329 (15.070) | 6.498 (10.411) | 7.879 (13.035) |
| SE | 2.070 | 2.298 | 1.626 | 1.166 |
| Median (range) | 8.981 (-17.961, 38.916) | 5.987 (-29.935, 35.923) | 5.987 (-11.974, 32.929) | 5.987 (-29.935, 38.916) |
| 95% CI | [3.556, 11.922] | [4.691, 13.967] | [3.212, 9.784] | [5.571, 10.187] |
|  |  |  |  |  |
| **Week 12** |  |  |  |  |
| *Observed* |  |  |  |  |
| n | 44 | 42 | 41 | 127 |
| Mean (SD) | 54.540 (11.578) | 52.525 (12.859) | 50.421 (10.817) | 52.544 (11.813) |
| SE | 1.745 | 1.984 | 1.689 | 1.048 |
| Median (range) | 54.948 (22.019, 69.916) | 50.458 (25.012, 69.916) | 48.961 (28.006, 69.916) | 51.954 (22.019, 69.916) |
| 95% CI | [51.020, 58.060] | [48.518, 56.532] | [47.007, 53.835] | [50.469, 54.618] |
| *Change from baseline* |  |  |  |  |
| n | 42 | 41 | 39 | 122 |
| Mean (SD) | 12.117 (14.680) | 12.558 (12.634) | 7.138 (11.044) | 10.674 (13.040) |
| SE | 2.265 | 1.973 | 1.768 | 1.181 |
| Median (range) | 11.974 (-23.948, 41.910) | 11.974 (-14.968, 38.916) | 5.987 (-8.981, 38.916) | 8.981 (-23.948, 41.910) |
| 95% CI | [7.542, 16.691] | [8.571, 16.546] | [3.558, 10.719] | [8.336, 13.011] |
|  |  |  |  |  |
| **Week 24** |  |  |  |  |
| *Observed* |  |  |  |  |
| n | 39 | 44 | 42 | 125 |
| Mean (SD) | 52.722 (14.019) | 51.682 (12.499) | 50.885 (12.224) | 51.739 (12.820) |
| SE | 2.245 | 1.884 | 1.886 | 1.147 |
| Median (range) | 51.954 (22.019, 69.916) | 50.458 (22.019, 69.916) | 51.954 (22.019, 69.916) | 51.954 (22.019, 69.916) |
| 95% CI | [48.177, 57.266] | [47.882, 55.482] | [47.076, 54.694] | [49.469, 54.008] |
| *Change from baseline* |  |  |  |  |
| n | 37 | 43 | 39 | 119 |
| Mean (SD) | 11.165 (15.724) | 11.626 (12.552) | 6.678 (11.481) | 9.861 (13.378) |
| SE | 2.585 | 1.914 | 1.838 | 1.226 |
| Median (range) | 8.981 (-20.955, 47.897) | 8.981 (-11.974, 38.916) | 8.981 (-11.974, 32.929) | 8.981 (-20.955, 47.897) |
| 95% CI | [5.922, 16.408] | [7.763, 15.489] | [2.956, 10.400] | [7.433, 12.290] |

Abbreviations: CI, confidence interval; NMV/r, nirmatrelvir-ritonavir; SD, standard deviation; SE, standard error; SF-36, 36-Item Short Form Health Survey.

### Supplementary Table 7. SF-36 Social Functioning scores – overall sample (N=150)

| Visit | 5-day NMV/r (N=52) | 10-day NMV/r (N=48) | 15-day NMV/r (N=50) | All arms (N=150) |
| --- | --- | --- | --- | --- |
| **Baseline (Day 1)** |  |  |  |  |
| *Observed* |  |  |  |  |
| n | 48 | 46 | 46 | 140 |
| Mean (SD) | 37.806 (13.402) | 34.776 (13.437) | 34.308 (12.838) | 35.661 (13.229) |
| SE | 1.934 | 1.981 | 1.893 | 1.118 |
| Median (range) | 34.893 (13.383, 56.403) | 34.893 (13.383, 56.403) | 34.893 (13.383, 56.403) | 34.893 (13.383, 56.403) |
| 95% CI | [33.914, 41.697] | [30.786, 38.767] | [30.496, 38.121] | [33.451, 37.872] |
|  |  |  |  |  |
| **Day 10** |  |  |  |  |
| *Observed* |  |  |  |  |
| n | 46 | 46 | 43 | 135 |
| Mean (SD) | 41.206 (10.714) | 41.323 (12.347) | 41.771 (12.270) | 41.426 (11.704) |
| SE | 1.580 | 1.821 | 1.871 | 1.007 |
| Median (range) | 40.271 (18.760, 56.403) | 40.271 (13.383, 56.403) | 45.648 (13.383, 56.403) | 40.271 (13.383, 56.403) |
| 95% CI | [38.024, 44.387] | [37.656, 44.989] | [37.995, 45.547] | [39.433, 43.418] |
| *Change from baseline* |  |  |  |  |
| n | 44 | 45 | 39 | 128 |
| Mean (SD) | 3.300 (14.817) | 6.692 (14.645) | 5.515 (14.466) | 5.168 (14.605) |
| SE | 2.234 | 2.183 | 2.316 | 1.291 |
| Median (range) | 0.000 (-37.643, 37.643) | 5.378 (-32.266, 37.643) | 5.378 (-21.510, 43.021) | 5.378 (-37.643, 43.021) |
| 95% CI | [-1.205, 7.805] | [2.292, 11.092] | [0.826, 10.205] | [2.613, 7.722] |
|  |  |  |  |  |
| **Day 21** |  |  |  |  |
| *Observed* |  |  |  |  |
| n | 42 | 42 | 43 | 127 |
| Mean (SD) | 44.368 (11.442) | 48.721 (10.633) | 46.023 (11.341) | 46.368 (11.202) |
| SE | 1.766 | 1.641 | 1.729 | 0.994 |
| Median (range) | 45.648 (13.383, 56.403) | 56.403 (18.760, 56.403) | 51.026 (18.760, 56.403) | 51.026 (13.383, 56.403) |
| 95% CI | [40.802, 47.933] | [45.408, 52.034] | [42.533, 49.514] | [44.401, 48.335] |
| *Change from baseline* |  |  |  |  |
| n | 40 | 41 | 40 | 121 |
| Mean (SD) | 5.915 (16.420) | 13.116 (14.827) | 11.024 (13.667) | 10.044 (15.193) |
| SE | 2.596 | 2.316 | 2.161 | 1.381 |
| Median (range) | 5.378 (-21.510, 43.021) | 16.133 (-21.510, 43.021) | 10.755 (-21.510, 43.021) | 10.755 (-21.510, 43.021) |
| 95% CI | [0.664, 11.167] | [8.436, 17.796] | [6.653, 15.395] | [7.309, 12.779] |
|  |  |  |  |  |
| **Day 44** |  |  |  |  |
| *Observed* |  |  |  |  |
| n | 43 | 44 | 44 | 131 |
| Mean (SD) | 49.150 (10.356) | 44.304 (12.658) | 47.115 (11.085) | 46.839 (11.501) |
| SE | 1.579 | 1.908 | 1.671 | 1.005 |
| Median (range) | 56.403 (13.383, 56.403) | 45.648 (13.383, 56.403) | 51.026 (18.760, 56.403) | 51.026 (13.383, 56.403) |
| 95% CI | [45.963, 52.337] | [40.455, 48.152] | [43.744, 50.485] | [44.851, 48.827] |
| *Change from baseline* |  |  |  |  |
| n | 41 | 43 | 41 | 125 |
| Mean (SD) | 10.624 (16.155) | 9.380 (16.887) | 12.067 (14.012) | 10.669 (15.663) |
| SE | 2.523 | 2.575 | 2.188 | 1.401 |
| Median (range) | 10.755 (-21.510, 43.021) | 10.755 (-43.021, 43.021) | 16.133 (-21.510, 43.021) | 10.755 (-43.021, 43.021) |
| 95% CI | [5.525, 15.723] | [4.182, 14.577] | [7.644, 16.489] | [7.896, 13.442] |
|  |  |  |  |  |
| **Week 12** |  |  |  |  |
| *Observed* |  |  |  |  |
| n | 44 | 42 | 41 | 127 |
| Mean (SD) | 47.970 (9.936) | 47.313 (10.522) | 47.353 (10.020) | 47.554 (10.084) |
| SE | 1.498 | 1.624 | 1.565 | 0.895 |
| Median (range) | 53.715 (29.515, 56.403) | 51.026 (24.138, 56.403) | 51.026 (24.138, 56.403) | 51.026 (24.138, 56.403) |
| 95% CI | [44.950, 50.991] | [44.034, 50.592] | [44.191, 50.516] | [45.783, 49.324] |
| *Change from baseline* |  |  |  |  |
| n | 42 | 41 | 39 | 122 |
| Mean (SD) | 9.347 (16.630) | 13.641 (16.492) | 12.823 (14.153) | 11.901 (15.815) |
| SE | 2.566 | 2.576 | 2.266 | 1.432 |
| Median (range) | 8.066 (-26.888, 43.021) | 16.133 (-21.510, 43.021) | 10.755 (-10.755, 43.021) | 10.755 (-26.888, 43.021) |
| 95% CI | [4.164, 14.529] | [8.435, 18.846] | [8.235, 17.412] | [9.067, 14.736] |
|  |  |  |  |  |
| **Week 24** |  |  |  |  |
| *Observed* |  |  |  |  |
| n | 39 | 44 | 42 | 125 |
| Mean (SD) | 47.716 (12.313) | 46.504 (12.325) | 46.032 (12.365) | 46.724 (12.255) |
| SE | 1.972 | 1.858 | 1.908 | 1.096 |
| Median (range) | 56.403 (13.383, 56.403) | 51.026 (13.383, 56.403) | 51.026 (13.383, 56.403) | 51.026 (13.383, 56.403) |
| 95% CI | [43.725, 51.708] | [42.756, 50.251] | [42.179, 49.886] | [44.554, 48.893] |
| *Change from baseline* |  |  |  |  |
| n | 37 | 43 | 39 | 119 |
| Mean (SD) | 11.337 (17.826) | 12.631 (16.960) | 11.307 (12.986) | 11.795 (15.938) |
| SE | 2.931 | 2.586 | 2.079 | 1.461 |
| Median (range) | 5.378 (-21.510, 43.021) | 16.133 (-26.888, 43.021) | 10.755 (-10.755, 43.021) | 10.755 (-26.888, 43.021) |
| 95% CI | [5.393, 17.280] | [7.412, 17.851] | [7.097, 15.516] | [8.901, 14.688] |

Abbreviations: CI, confidence interval; NMV/r, nirmatrelvir-ritonavir; SD, standard deviation; SE, standard error; SF-36, 36-Item Short Form Health Survey.

### Supplementary Table 8. SF-36 Role-Emotional scores – overall sample (N=150)

| Visit | 5-day NMV/r (N=52) | 10-day NMV/r (N=48) | 15-day NMV/r (N=50) | All arms (N=150) |
| --- | --- | --- | --- | --- |
| **Baseline (Day 1)** |  |  |  |  |
| *Observed* |  |  |  |  |
| n | 48 | 46 | 46 | 140 |
| Mean (SD) | 38.406 (11.341) | 36.256 (12.508) | 37.490 (11.445) | 37.399 (11.719) |
| SE | 1.637 | 1.844 | 1.687 | 0.990 |
| Median (range) | 40.535 (17.821, 55.679) | 36.750 (10.249, 55.679) | 36.750 (10.249, 55.679) | 40.535 (10.249, 55.679) |
| 95% CI | [35.113, 41.699] | [32.542, 39.970] | [34.092, 40.889] | [35.440, 39.357] |
|  |  |  |  |  |
| **Day 10** |  |  |  |  |
| *Observed* |  |  |  |  |
| n | 46 | 46 | 43 | 135 |
| Mean (SD) | 41.605 (9.633) | 42.675 (9.698) | 42.208 (9.750) | 42.162 (9.630) |
| SE | 1.420 | 1.430 | 1.487 | 0.829 |
| Median (range) | 44.321 (21.607, 55.679) | 44.321 (14.035, 55.679) | 44.321 (17.821, 55.679) | 44.321 (14.035, 55.679) |
| 95% CI | [38.745, 44.466] | [39.795, 45.555] | [39.208, 45.209] | [40.523, 43.801] |
| *Change from baseline* |  |  |  |  |
| n | 44 | 45 | 39 | 128 |
| Mean (SD) | 3.700 (11.930) | 6.478 (11.056) | 3.106 (8.894) | 4.496 (10.789) |
| SE | 1.798 | 1.648 | 1.424 | 0.954 |
| Median (range) | 3.786 (-34.072, 30.286) | 3.786 (-11.357, 34.072) | 0.000 (-15.143, 26.501) | 3.786 (-34.072, 34.072) |
| 95% CI | [0.073, 7.327] | [3.156, 9.799] | [0.223, 5.989] | [2.609, 6.383] |
|  |  |  |  |  |
| **Day 21** |  |  |  |  |
| *Observed* |  |  |  |  |
| n | 42 | 42 | 43 | 127 |
| Mean (SD) | 46.304 (8.618) | 48.377 (9.021) | 46.434 (10.262) | 47.034 (9.309) |
| SE | 1.330 | 1.392 | 1.565 | 0.826 |
| Median (range) | 48.107 (25.392, 55.679) | 51.893 (17.821, 55.679) | 51.893 (21.607, 55.679) | 51.893 (17.821, 55.679) |
| 95% CI | [43.619, 48.990] | [45.566, 51.189] | [43.276, 49.592] | [45.399, 48.669] |
| *Change from baseline* |  |  |  |  |
| n | 40 | 41 | 40 | 121 |
| Mean (SD) | 7.666 (12.874) | 11.819 (12.788) | 8.045 (8.624) | 9.199 (11.667) |
| SE | 2.035 | 1.997 | 1.364 | 1.061 |
| Median (range) | 7.572 (-18.929, 34.072) | 7.572 (-7.572, 45.429) | 7.572 (-7.572, 26.501) | 7.572 (-18.929, 45.429) |
| 95% CI | [3.549, 11.783] | [7.783, 15.855] | [5.287, 10.803] | [7.099, 11.298] |
|  |  |  |  |  |
| **Day 44** |  |  |  |  |
| *Observed* |  |  |  |  |
| n | 43 | 44 | 44 | 131 |
| Mean (SD) | 47.315 (8.726) | 47.161 (12.466) | 48.021 (9.504) | 47.500 (10.296) |
| SE | 1.331 | 1.879 | 1.433 | 0.900 |
| Median (range) | 48.107 (21.607, 55.679) | 51.893 (10.249, 55.679) | 51.893 (25.392, 55.679) | 51.893 (10.249, 55.679) |
| 95% CI | [44.629, 50.000] | [43.371, 50.951] | [45.132, 50.910] | [45.720, 49.280] |
| *Change from baseline* |  |  |  |  |
| n | 41 | 43 | 41 | 125 |
| Mean (SD) | 9.234 (12.867) | 10.301 (15.573) | 9.603 (10.091) | 9.722 (12.984) |
| SE | 2.010 | 2.375 | 1.576 | 1.161 |
| Median (range) | 7.572 (-15.143, 37.858) | 11.357 (-37.858, 45.429) | 7.572 (-7.572, 30.286) | 7.572 (-37.858, 45.429) |
| 95% CI | [5.172, 13.295] | [5.508, 15.093] | [6.418, 12.788] | [7.423, 12.021] |
|  |  |  |  |  |
| **Week 12** |  |  |  |  |
| *Observed* |  |  |  |  |
| n | 44 | 42 | 41 | 127 |
| Mean (SD) | 49.054 (8.645) | 48.918 (9.142) | 48.292 (8.505) | 48.763 (8.705) |
| SE | 1.303 | 1.411 | 1.328 | 0.772 |
| Median (range) | 53.786 (29.178, 55.679) | 51.893 (21.607, 55.679) | 51.893 (29.178, 55.679) | 51.893 (21.607, 55.679) |
| 95% CI | [46.425, 51.682] | [46.070, 51.767] | [45.607, 50.976] | [47.234, 50.292] |
| *Change from baseline* |  |  |  |  |
| n | 42 | 41 | 39 | 122 |
| Mean (SD) | 10.186 (12.274) | 13.481 (13.225) | 10.193 (9.279) | 11.295 (11.762) |
| SE | 1.894 | 2.065 | 1.486 | 1.065 |
| Median (range) | 7.572 (-15.143, 34.072) | 11.357 (-7.572, 45.429) | 7.572 (-3.786, 30.286) | 11.357 (-15.143, 45.429) |
| 95% CI | [6.361, 14.010] | [9.307, 17.655] | [7.184, 13.201] | [9.187, 13.403] |
|  |  |  |  |  |
| **Week 24** |  |  |  |  |
| *Observed* |  |  |  |  |
| n | 39 | 44 | 42 | 125 |
| Mean (SD) | 48.010 (9.845) | 49.140 (9.604) | 47.025 (12.494) | 48.077 (10.681) |
| SE | 1.576 | 1.448 | 1.928 | 0.955 |
| Median (range) | 51.893 (21.607, 55.679) | 51.893 (21.607, 55.679) | 51.893 (10.249, 55.679) | 51.893 (10.249, 55.679) |
| 95% CI | [44.819, 51.201] | [46.220, 52.059] | [43.132, 50.919] | [46.186, 49.968] |
| *Change from baseline* |  |  |  |  |
| n | 37 | 43 | 39 | 119 |
| Mean (SD) | 9.311 (13.955) | 13.118 (13.304) | 8.251 (11.452) | 10.339 (13.009) |
| SE | 2.294 | 2.029 | 1.834 | 1.193 |
| Median (range) | 7.572 (-18.929, 37.858) | 11.357 (-11.357, 45.429) | 7.572 (-22.715, 34.072) | 7.572 (-22.715, 45.429) |
| 95% CI | [4.658, 13.964] | [9.024, 17.213] | [4.539, 11.963] | [7.978, 12.701] |

Abbreviations: CI, confidence interval; NMV/r, nirmatrelvir-ritonavir; SD, standard deviation; SE, standard error; SF-36, 36-Item Short Form Health Survey.

### Supplementary Table 9. SF-36 Mental Health scores – overall sample (N=150)

| Visit | 5-day NMV/r (N=52) | 10-day NMV/r (N=48) | 15-day NMV/r (N=50) | All arms (N=150) |
| --- | --- | --- | --- | --- |
| **Baseline (Day 1)** |  |  |  |  |
| *Observed* |  |  |  |  |
| n | 48 | 46 | 46 | 140 |
| Mean (SD) | 46.000 (11.795) | 43.195 (12.194) | 43.074 (12.448) | 44.117 (12.133) |
| SE | 1.702 | 1.798 | 1.835 | 1.025 |
| Median (range) | 46.808 (21.874, 63.431) | 44.038 (8.022, 63.431) | 41.267 (16.333, 63.431) | 44.038 (8.022, 63.431) |
| 95% CI | [42.575, 49.425] | [39.573, 46.816] | [39.378, 46.771] | [42.089, 46.144] |
|  |  |  |  |  |
| **Day 10** |  |  |  |  |
| *Observed* |  |  |  |  |
| n | 46 | 46 | 43 | 135 |
| Mean (SD) | 49.398 (10.274) | 47.411 (12.928) | 48.355 (10.650) | 48.388 (11.305) |
| SE | 1.515 | 1.906 | 1.624 | 0.973 |
| Median (range) | 52.349 (30.185, 63.431) | 52.349 (16.333, 63.431) | 49.579 (27.415, 63.431) | 49.579 (16.333, 63.431) |
| 95% CI | [46.347, 52.449] | [43.571, 51.250] | [45.077, 51.632] | [46.464, 50.313] |
| *Change from baseline* |  |  |  |  |
| n | 44 | 45 | 39 | 128 |
| Mean (SD) | 2.833 (11.090) | 4.371 (11.717) | 3.481 (8.425) | 3.571 (10.529) |
| SE | 1.672 | 1.747 | 1.349 | 0.931 |
| Median (range) | 0.000 (-27.705, 27.705) | 2.770 (-33.246, 38.787) | 2.770 (-11.082, 30.475) | 2.770 (-33.246, 38.787) |
| 95% CI | [-0.538, 6.205] | [0.851, 7.891] | [0.750, 6.212] | [1.730, 5.413] |
|  |  |  |  |  |
| **Day 21** |  |  |  |  |
| *Observed* |  |  |  |  |
| n | 42 | 42 | 43 | 127 |
| Mean (SD) | 50.436 (11.208) | 51.755 (9.971) | 48.290 (11.170) | 50.146 (10.811) |
| SE | 1.729 | 1.539 | 1.703 | 0.959 |
| Median (range) | 55.120 (21.874, 63.431) | 55.120 (27.415, 63.431) | 46.808 (21.874, 63.431) | 52.349 (21.874, 63.431) |
| 95% CI | [46.944, 53.929] | [48.648, 54.863] | [44.853, 51.728] | [48.247, 52.044] |
| *Change from baseline* |  |  |  |  |
| n | 40 | 41 | 40 | 121 |
| Mean (SD) | 4.433 (13.942) | 8.244 (11.664) | 4.086 (9.220) | 5.610 (11.822) |
| SE | 2.204 | 1.822 | 1.458 | 1.075 |
| Median (range) | 2.770 (-33.246, 38.787) | 5.541 (-24.934, 49.869) | 2.770 (-16.623, 22.164) | 5.541 (-33.246, 49.869) |
| 95% CI | [-0.026, 8.891] | [4.562, 11.925] | [1.138, 7.035] | [3.482, 7.737] |
|  |  |  |  |  |
| **Day 44** |  |  |  |  |
| *Observed* |  |  |  |  |
| n | 43 | 44 | 44 | 131 |
| Mean (SD) | 52.220 (10.312) | 48.508 (12.970) | 48.823 (11.436) | 49.832 (11.666) |
| SE | 1.573 | 1.955 | 1.724 | 1.019 |
| Median (range) | 55.120 (21.874, 63.431) | 52.349 (21.874, 63.431) | 49.579 (30.185, 63.431) | 52.349 (21.874, 63.431) |
| 95% CI | [49.047, 55.394] | [44.565, 52.452] | [45.346, 52.300] | [47.816, 51.849] |
| *Change from baseline* |  |  |  |  |
| n | 41 | 43 | 41 | 125 |
| Mean (SD) | 6.555 (14.083) | 5.541 (14.153) | 4.189 (9.295) | 5.430 (12.673) |
| SE | 2.199 | 2.158 | 1.452 | 1.134 |
| Median (range) | 2.770 (-16.623, 38.787) | 5.541 (-24.934, 55.409) | 2.770 (-8.311, 27.705) | 2.770 (-24.934, 55.409) |
| 95% CI | [2.110, 11.000] | [1.185, 9.896] | [1.256, 7.123] | [3.187, 7.674] |
|  |  |  |  |  |
| **Week 12** |  |  |  |  |
| *Observed* |  |  |  |  |
| n | 44 | 42 | 41 | 127 |
| Mean (SD) | 52.538 (10.199) | 50.502 (11.812) | 50.187 (10.793) | 51.106 (10.909) |
| SE | 1.538 | 1.823 | 1.686 | 0.968 |
| Median (range) | 55.120 (30.185, 63.431) | 55.120 (24.644, 63.431) | 52.349 (24.644, 63.431) | 52.349 (24.644, 63.431) |
| 95% CI | [49.437, 55.639] | [46.821, 54.183] | [46.780, 53.593] | [49.190, 53.021] |
| *Change from baseline* |  |  |  |  |
| n | 42 | 41 | 39 | 122 |
| Mean (SD) | 7.190 (14.416) | 8.379 (12.068) | 6.180 (11.466) | 7.267 (12.675) |
| SE | 2.224 | 1.885 | 1.836 | 1.148 |
| Median (range) | 5.541 (-22.164, 38.787) | 5.541 (-19.393, 41.557) | 2.770 (-19.393, 30.475) | 5.541 (-22.164, 41.557) |
| 95% CI | [2.698, 11.682] | [4.570, 12.188] | [2.463, 9.897] | [4.995, 9.539] |
|  |  |  |  |  |
| **Week 24** |  |  |  |  |
| *Observed* |  |  |  |  |
| n | 39 | 44 | 42 | 125 |
| Mean (SD) | 52.704 (11.158) | 50.586 (11.633) | 49.447 (12.145) | 50.864 (11.646) |
| SE | 1.787 | 1.754 | 1.874 | 1.042 |
| Median (range) | 57.890 (21.874, 63.431) | 52.349 (16.333, 63.431) | 52.349 (16.333, 63.431) | 52.349 (16.333, 63.431) |
| 95% CI | [49.087, 56.321] | [47.049, 54.123] | [45.662, 53.231] | [48.803, 52.926] |
| *Change from baseline* |  |  |  |  |
| n | 37 | 43 | 39 | 119 |
| Mean (SD) | 7.188 (16.305) | 8.054 (12.708) | 4.546 (10.739) | 6.635 (13.342) |
| SE | 2.681 | 1.938 | 1.720 | 1.223 |
| Median (range) | 5.541 (-33.246, 38.787) | 5.541 (-19.393, 36.016) | 2.770 (-22.164, 27.705) | 5.541 (-33.246, 38.787) |
| 95% CI | [1.752, 12.625] | [4.143, 11.965] | [1.065, 8.028] | [4.213, 9.057] |

Abbreviations: CI, confidence interval; NMV/r, nirmatrelvir-ritonavir; SD, standard deviation; SE, standard error; SF-36, 36-Item Short Form Health Survey.

### Supplementary Table 10. SF-36 PCS scores – overall sample (N=150)

| Visit | 5-day NMV/r (N=52) | 10-day NMV/r (N=48) | 15-day NMV/r (N=50) | All arms (N=150) |
| --- | --- | --- | --- | --- |
| **Baseline (Day 1)** |  |  |  |  |
| *Observed* |  |  |  |  |
| n | 48 | 46 | 46 | 140 |
| Mean (SD) | 36.871 (8.275) | 37.817 (8.400) | 39.004 (8.548) | 37.883 (8.392) |
| SE | 1.194 | 1.238 | 1.260 | 0.709 |
| Median (range) | 37.911 (18.127, 58.866) | 39.088 (16.910, 54.466) | 37.841 (20.896, 56.744) | 38.178 (16.910, 58.866) |
| 95% CI | [34.468, 39.274] | [35.323, 40.311] | [36.465, 41.542] | [36.480, 39.285] |
|  |  |  |  |  |
| **Day 10** |  |  |  |  |
| *Observed* |  |  |  |  |
| n | 46 | 46 | 43 | 135 |
| Mean (SD) | 41.880 (9.582) | 45.372 (8.246) | 43.935 (10.278) | 43.725 (9.428) |
| SE | 1.413 | 1.216 | 1.567 | 0.811 |
| Median (range) | 41.404 (14.089, 63.584) | 44.483 (25.484, 59.189) | 45.588 (23.208, 62.369) | 43.341 (14.089, 63.584) |
| *Change from baseline* |  |  |  |  |
| n | 44 | 45 | 39 | 128 |
| Mean (SD) | 4.788 (8.130) | 7.597 (8.272) | 4.841 (7.808) | 5.792 (8.131) |
| SE | 1.226 | 1.233 | 1.250 | 0.709 |
| Median (range) | 4.538 (-18.165, 22.822) | 7.604 (-7.129, 29.913) | 4.447 (-7.264, 20.867) | 5.015 (-18.165, 29.913) |
| 95% CI | [2.317, 7.260] | [5.112, 10.082] | [2.310, 7.372] | [4.370, 7.214] |
|  |  |  |  |  |
| **Day 21** |  |  |  |  |
| *Observed* |  |  |  |  |
| n | 42 | 42 | 43 | 127 |
| Mean (SD) | 44.450 (10.940) | 47.767 (9.999) | 48.265 (8.383) | 46.839 (9.889) |
| SE | 1.688 | 1.543 | 1.278 | 0.878 |
| Median (range) | 43.956 (11.658, 62.198) | 51.137 (23.221, 60.359) | 48.268 (29.503, 63.746) | 46.633 (11.658, 63.746) |
| 95% CI | [41.041, 47.859] | [44.651, 50.883] | [45.685, 50.844] | [45.102, 48.575] |
| *Change from baseline* |  |  |  |  |
| n | 40 | 41 | 40 | 121 |
| Mean (SD) | 6.856 (8.065) | 9.268 (9.496) | 9.870 (8.583) | 8.669 (8.766) |
| SE | 1.275 | 1.483 | 1.357 | 0.797 |
| Median (range) | 8.625 (-7.114, 24.204) | 7.977 (-10.210, 34.143) | 10.252 (-9.455, 33.352) | 8.536 (-10.210, 34.143) |
| 95% CI | [4.276, 9.435] | [6.270, 12.265] | [7.125, 12.615] | [7.092, 10.247] |
|  |  |  |  |  |
| **Day 44** |  |  |  |  |
| *Observed* |  |  |  |  |
| n | 43 | 44 | 44 | 131 |
| Mean (SD) | 47.181 (9.019) | 46.762 (11.632) | 47.639 (9.273) | 47.194 (9.980) |
| SE | 1.375 | 1.754 | 1.398 | 0.872 |
| Median (range) | 49.171 (29.842, 60.700) | 49.750 (20.564, 62.082) | 50.220 (24.428, 60.880) | 50.139 (20.564, 62.082) |
| 95% CI | [44.405, 49.956] | [43.226, 50.299] | [44.820, 50.459] | [45.469, 48.919] |
| *Change from baseline* |  |  |  |  |
| n | 41 | 43 | 41 | 125 |
| Mean (SD) | 9.313 (9.552) | 8.685 (11.839) | 9.070 (9.002) | 9.017 (10.154) |
| SE | 1.492 | 1.805 | 1.406 | 0.908 |
| Median (range) | 9.342 (-7.736, 29.756) | 8.912 (-20.888, 36.234) | 7.589 (-10.226, 32.901) | 8.706 (-20.888, 36.234) |
| 95% CI | [6.298, 12.328] | [5.041, 12.328] | [6.229, 11.912] | [7.220, 10.815] |
|  |  |  |  |  |
| **Week 12** |  |  |  |  |
| *Observed* |  |  |  |  |
| n | 44 | 42 | 41 | 127 |
| Mean (SD) | 49.557 (8.368) | 47.606 (10.057) | 47.738 (10.920) | 48.325 (9.771) |
| SE | 1.261 | 1.552 | 1.705 | 0.867 |
| Median (range) | 51.738 (27.797, 61.138) | 49.499 (23.835, 62.361) | 50.707 (21.644, 65.502) | 50.969 (21.644, 65.502) |
| 95% CI | [47.013, 52.101] | [44.472, 50.740] | [44.292, 51.185] | [46.609, 50.041] |
| *Change from baseline* |  |  |  |  |
| n | 42 | 41 | 39 | 122 |
| Mean (SD) | 11.819 (9.939) | 10.205 (10.307) | 10.287 (8.973) | 10.287 (8.973) |
| SE | 1.534 | 1.610 | 1.522 | 0.894 |
| Median (range) | 12.836 (-11.099, 30.801) | 9.292 (-8.858, 35.587) | 9.187 (-8.345, 32.247) | 10.587 (-11.099, 35.587) |
| 95% CI | [8.084, 14.278] | [6.952, 13.458] | [6.331, 12.492] | [8.518, 12.057] |
|  |  |  |  |  |
| **Week 24** |  |  |  |  |
| *Observed* |  |  |  |  |
| n | 39 | 44 | 42 | 125 |
| Mean (SD) | 47.462 (11.047) | 48.023 (10.657) | 48.063 (10.476) | 47.861 (10.637) |
| SE | 1.769 | 1.607 | 1.616 | 0.951 |
| Median (range) | 51.211 (22.502, 62.806) | 50.159 (18.612, 65.502) | 49.584 (23.659, 60.682) | 49.936 (18.612, 65.502) |
| 95% CI | [43.881, 51.043] | [44.783, 51.263] | [44.798, 51.327] | [45.978, 49.744] |
| *Change from baseline* |  |  |  |  |
| n | 37 | 43 | 39 | 119 |
| Mean (SD) | 9.747 (11.449) | 10.560 (10.273) | 9.221 (11.308) | 9.868 (10.911) |
| SE | 1.882 | 1.567 | 1.811 | 1.000 |
| Median (range) | 9.827 (-15.696, 30.944) | 9.247 (-7.792, 27.485) | 11.019 (-18.517, 34.072) | 9.827 (-18.517, 34.072) |
| 95% CI | [5.929, 13.564] | [7.399, 13.721] | [5.555, 12.887] | [7.888, 11.849] |

Abbreviations: CI, confidence interval; NMV/r, nirmatrelvir-ritonavir; PCS, Physical Component Summary; SD, standard deviation; SE, standard error; SF-36, 36-Item Short Form Health Survey.

### Supplementary Table 11. SF-36 MCS scores – overall sample (N=150)

| Visit | 5-day NMV/r (N=52) | 10-day NMV/r (N=48) | 15-day NMV/r (N=50) | All arms (N=150) |
| --- | --- | --- | --- | --- |
| **Baseline (Day 1)** |  |  |  |  |
| *Observed* |  |  |  |  |
| n | 48 | 46 | 46 | 140 |
| Mean (SD) | 43.872 (10.773) | 39.965 (11.543) | 40.580 (12.428) | 41.507 (11.635) |
| SE | 1.555 | 1.702 | 1.832 | 0.983 |
| Median (range) | 45.145 (18.846, 67.276) | 39.438 (10.963, 61.921) | 39.733 (11.235, 65.606) | 41.294 (10.963, 67.276) |
| 95% CI | [40.744, 47.000] | [36.537, 43.393] | [36.890, 44.271] | [39.563, 43.451] |
|  |  |  |  |  |
| **Day 10** |  |  |  |  |
| *Observed* |  |  |  |  |
| n | 46 | 46 | 43 | 135 |
| Mean (SD) | 45.947 (9.902) | 44.180 (11.453) | 45.703 (10.776) | 45.267 (10.677) |
| SE | 1.460 | 1.689 | 1.643 | 0.919 |
| Median (range) | 46.474 (28.433, 68.190) | 45.082 (15.257, 59.488) | 46.482 (25.535, 66.272) | 45.647 (15.257, 68.190) |
| 95% CI | [43.007, 48.888] | [40.779, 47.581] | [42.386, 49.019] | [43.450, 47.085] |
| *Change from baseline* |  |  |  |  |
| n | 44 | 45 | 39 | 128 |
| Mean (SD) | 2.008 (11.156) | 4.233 (11.157) | 2.971 (9.803) | 3.084 (10.719) |
| SE | 1.682 | 1.663 | 1.570 | 0.947 |
| Median (range) | 1.707 (-22.346, 29.381) | 4.370 (-32.069, 28.724) | 0.205 (-11.221, 29.868) | 2.853 (-32.069, 29.868) |
| 95% CI | [-1.384, 5.400] | [0.881, 7.584] | [-0.207, 6.149] | [1.209, 4.958] |
|  |  |  |  |  |
| **Day 21** |  |  |  |  |
| *Observed* |  |  |  |  |
| n | 42 | 42 | 43 | 127 |
| Mean (SD) | 48.505 (11.013) | 50.738 (9.762) | 47.299 (11.646) | 48.835 (10.850) |
| SE | 1.699 | 1.506 | 1.776 | 0.963 |
| Median (range) | 51.714 (20.251, 68.366) | 53.780 (24.247, 61.767) | 48.681 (18.594, 64.799) | 52.439 (18.594, 68.366) |
| 95% CI | [45.073, 51.937] | [47.696, 53.780] | [43.715, 50.883] | [46.930, 50.740] |
| *Change from baseline* |  |  |  |  |
| n | 40 | 41 | 40 | 121 |
| Mean (SD) | 4.637 (14.380) | 10.453 (12.008) | 5.215 (9.097) | 6.799 (12.212) |
| SE | 2.274 | 1.875 | 1.438 | 1.110 |
| Median (range) | 3.469 (-32.915, 32.666) | 10.536 (-26.451, 41.814) | 3.627 (-8.024, 26.089) | 5.211 (-32.915, 41.814) |
| 95% CI | [0.038, 9.235] | [6.663, 14.244] | [2.306, 8.125] | [4.601, 8.997] |
|  |  |  |  |  |
| **Day 44** |  |  |  |  |
| *Observed* |  |  |  |  |
| n | 43 | 44 | 44 | 131 |
| Mean (SD) | 50.545 (9.905) | 47.615 (12.349) | 48.668 (11.347) | 48.930 (11.233) |
| SE | 1.510 | 1.862 | 1.711 | 0.981 |
| Median (range) | 52.762 (21.214, 61.841) | 52.094 (18.267, 61.818) | 52.250 (24.973, 64.472) | 52.441 (18.267, 64.472) |
| 95% CI | [47.497, 53.593] | [43.861, 51.369] | [45.218, 52.117] | [46.989, 50.872] |
| *Change from baseline* |  |  |  |  |
| n | 41 | 43 | 41 | 125 |
| Mean (SD) | 7.065 (13.510) | 7.550 (14.893) | 6.388 (10.352) | 7.010 (12.992) |
| SE | 2.110 | 2.271 | 1.617 | 1.162 |
| Median (range) | 5.686 (-22.563, 32.506) | 7.984 (-31.919, 48.370) | 5.142 (-13.802, 29.541) | 7.087 (-31.919, 48.370) |
| 95% CI | [2.801, 11.329] | [2.967, 12.133] | [3.120, 9.655] | [4.709, 9.310] |
|  |  |  |  |  |
| **Week 12** |  |  |  |  |
| *Observed* |  |  |  |  |
| n | 44 | 42 | 41 | 127 |
| Mean (SD) | 51.227 (9.797) | 50.169 (10.345) | 49.275 (10.282) | 50.247 (10.089) |
| SE | 1.477 | 1.596 | 1.606 | 0.895 |
| Median (range) | 54.204 (27.695, 63.511) | 53.446 (25.411, 65.455) | 50.699 (24.859, 64.019) | 53.296 (24.859, 65.455) |
| 95% CI | [48.249, 54.206] | [46.945, 53.392] | [46.030, 52.521] | [48.475, 52.019] |
| *Change from baseline* |  |  |  |  |
| n | 42 | 41 | 39 | 122 |
| Mean (SD) | 7.623 (13.495) | 11.173 (12.235) | 7.723 (10.916) | 8.848 (12.303) |
| SE | 2.082 | 1.911 | 1.748 | 1.114 |
| Median (range) | 7.460 (-21.714, 39.409) | 8.821 (-14.651, 38.890) | 6.791 (-12.801, 33.526) | 7.736 (-21.714, 39.409) |
| 95% CI | [3.418, 11.828] | [7.312, 15.035] | [4.185, 11.261] | [6.643, 11.053] |
|  |  |  |  |  |
| **Week 24** |  |  |  |  |
| *Observed* |  |  |  |  |
| n | 39 | 44 | 42 | 125 |
| Mean (SD) | 51.195 (10.583) | 49.792 (11.037) | 48.192 (12.782) | 49.692 (11.492) |
| SE | 1.695 | 1.664 | 1.972 | 1.028 |
| Median (range) | 53.757 (22.177, 62.319) | 54.003 (22.921, 64.248) | 52.580 (12.683, 61.745) | 53.340 (12.683, 64.248) |
| 95% CI | [47.764, 54.625] | [46.437, 53.148] | [44.209, 52.175] | [47.658, 51.727] |
| *Change from baseline* |  |  |  |  |
| n | 37 | 43 | 39 | 119 |
| Mean (SD) | 8.166 (15.687) | 10.404 (12.393) | 5.901 (10.985) | 8.233 (13.119) |
| SE | 2.579 | 1.890 | 1.759 | 1.203 |
| Median (range) | 6.803 (-24.864, 35.210) | 7.928 (-18.761, 37.494) | 3.528 (-13.926, 31.357) | 6.630 (-24.864, 37.494) |
| 95% CI | [2.936, 13.397] | [6.590, 14.219] | [2.340, 9.462] | [5.851, 10.614] |

Abbreviations: CI, confidence interval; MCS, Mental Component Summary; NMV/r, nirmatrelvir-ritonavir; SD, standard deviation; SE, standard error; SF-36, 36-Item Short Form Health Survey.

### Supplementary Table 12. EQ-5D-5L completion rates

| **Time Point** | **5-day NMV/r** | **10-day NMV/r** | **15-day NMV/r** | **All arms** |
| --- | --- | --- | --- | --- |
| ***Overall sample*** | ***N=52*** | ***N=48*** | ***N=50*** | ***N=150*** |
| Baseline (Day 1) | 48 (92.3%) | 46 (95.8%) | 46 (92.0%) | 140 (93.3%) |
| Day 5 | 43 (82.7%) | 45 (93.8%) | 45 (90.0%) | 133 (88.7%) |
| Day 15 | 47 (90.4%) | 44 (91.7%) | 43 (86.0%) | 134 (89.3%) |
| Day 44 | 43 (82.7%) | 43 (89.6%) | 44 (88.0%) | 130 (86.7%) |
| Week 12 | 43 (82.7%) | 41 (85.4%) | 41 (82.0%) | 125 (83.3%) |
| Week 24 | 39 (75.0%) | 43 (89.6%) | 42 (84.0%) | 124 (82.7%) |
| ***Severe IC*** | ***N=20*** | ***N=17*** | ***N=20*** | ***N=57*** |
| Baseline (Day 1) | 19 (95.0%) | 16 (94.1%) | 18 (90.0%) | 53 (93.0%) |
| Day 5 | 14 (70.0%) | 15 (88.2%) | 17 (85.0%) | 46 (80.7%) |
| Day 15 | 17 (85.0%) | 15 (88.2%) | 15 (75.0%) | 47 (82.5%) |
| Day 44 | 15 (75.0%) | 15 (88.2%) | 15 (75.0%) | 45 (79.0%) |
| Week 12 | 13 (65.0%) | 12 (70.6%) | 13 (65.0%) | 38 (66.7%) |
| Week 24 | 13 (65.0%) | 14 (82.4%) | 13 (65.0%) | 40 (70.2%) |
| ***Non-severe IC*** | ***N=32*** | ***N=31*** | ***N=30*** | ***N=93*** |
| Baseline (Day 1) | 29 (90.6%) | 30 (96.8%) | 28 (93.3%) | 87 (93.5%) |
| Day 5 | 29 (90.6%) | 30 (96.8%) | 28 (93.3%) | 87 (93.5%) |
| Day 15 | 30 (93.8%) | 29 (93.5%) | 28 (93.3%) | 87 (93.5%) |
| Day 44 | 28 (87.5%) | 28 (90.3%) | 29 (96.7%) | 85 (91.4%) |
| Week 12 | 30 (93.8%) | 29 (93.5%) | 28 (93.3%) | 87 (93.5%) |
| Week 24 | 26 (81.3%) | 29 (93.5%) | 29 (96.7%) | 84 (90.3%) |

Abbreviations: IC, immunocompromise; NMV/r, nirmatrelvir-ritonavir.

### Supplementary Table 13. EQ-5D-5L Self-Care scores – overall sample (N=150)

|  | 5-day NMV/r (N=52) | 10-day NMV/r (N=48) | 15-day NMV/r (N=50) | All arms (N=150) |
| --- | --- | --- | --- | --- |
|  | n (%) | n (%) | n (%) | n (%) |
| **Baseline (Day 1)** |  |  |  |  |
| n | 48 (92.308) | 46 (95.833) | 46 (92.000) | 140 (93.333) |
| None | 38 (79.167) | 32 (69.565) | 29 (63.043) | 99 (70.714) |
| Slight | 4 (8.333) | 8 (17.391) | 12 (26.087) | 24 (17.143) |
| Moderate | 5 (10.417) | 5 (10.870) | 5 (10.870) | 15 (10.714) |
| Severe | 0 | 1 (2.174) | 0 | 1 (0.714) |
| Extreme/Unable | 1 (2.083) | 0 | 0 | 1 (0.714) |
|  |  |  |  |  |
| **Day 5** |  |  |  |  |
| n | 43 (82.692) | 45 (93.750) | 45 (90.000) | 133 (88.667) |
| None | 35 (81.395) | 37 (82.222) | 36 (80.000) | 108 (81.203) |
| Slight | 7 (16.279) | 6 (13.333) | 6 (13.333) | 19 (14.286) |
| Moderate | 1 (2.326) | 1 (2.222) | 3 (6.667) | 5 (3.759) |
| Severe | 0 | 1 (2.222) | 0 | 1 (0.752) |
| Extreme/Unable | 0 | 0 | 0 | 0 |
|  |  |  |  |  |
| **Day 15** |  |  |  |  |
| n | 47 (90.385) | 44 (91.667) | 43 (86.000) | 134 (89.333) |
| None | 41 (87.234) | 41 (93.182) | 38 (88.372) | 120 (89.552) |
| Slight | 4 (8.511) | 3 (6.818) | 4 (9.302) | 11 (8.209) |
| Moderate | 2 (4.255) | 0 | 1 (2.326) | 3 (2.239) |
| Severe | 0 | 0 | 0 | 0 |
| Extreme/Unable | 0 | 0 | 0 | 0 |
|  |  |  |  |  |
| **Day 44** |  |  |  |  |
| n | 43 (82.692) | 43 (89.583) | 44 (88.000) | 130 (86.667) |
| None | 39 (90.698) | 35 (81.395) | 38 (86.364) | 112 (86.154) |
| Slight | 4 (9.302) | 6 (13.953) | 5 (11.364) | 15 (11.538) |
| Moderate | 0 | 2 (4.651) | 1 (2.273) | 3 (2.308) |
| Severe | 0 | 0 | 0 | 0 |
| Extreme/Unable | 0 | 0 | 0 | 0 |
|  |  |  |  |  |
| **Week 12** |  |  |  |  |
| n | 43 (82.692) | 41 (85.417) | 41 (82.000) | 125 (83.333) |
| None | 37 (86.047) | 36 (87.805) | 35 (85.366) | 108 (86.400) |
| Slight | 3 (6.977) | 3 (7.317) | 4 (9.756) | 10 (8.000) |
| Moderate | 3 (6.977) | 1 (2.439) | 2 (4.878) | 6 (4.800) |
| Severe | 0 | 1 (2.439) | 0 | 1 (0.800) |
| Extreme/Unable | 0 | 0 | 0 | 0 |
|  |  |  |  |  |
| **Week 24** |  |  |  |  |
| n | 39 (75.000) | 43 (89.583) | 42 (84.000) | 124 (82.667) |
| None | 32 (82.051) | 36 (83.721) | 38 (90.476) | 106 (85.484) |
| Slight | 5 (12.821) | 7 (16.279) | 3 (7.143) | 15 (12.097) |
| Moderate | 2 (5.128) | 0 | 1 (2.381) | 3 (2.419) |
| Severe | 0 | 0 | 0 | 0 |
| Extreme/Unable | 0 | 0 | 0 | 0 |

Abbreviation: NMV/r, nirmatrelvir-ritonavir.

### Supplementary Table 14. EQ-5D-5L Mobility scores – overall sample (N=150)

|  | 5-day NMV/r (N=52) | 10-day NMV/r (N=48) | 15-day NMV/r (N=50) | All arms (N=150) |
| --- | --- | --- | --- | --- |
|  | n (%) | n (%) | n (%) | n (%) |
| **Baseline (Day 1)** |  |  |  |  |
| n (%) | 48 (92.308) | 46 (95.833) | 46 (92.000) | 140 (93.333) |
| None | 25 (52.083) | 24 (52.174) | 21 (45.652) | 70 (50.000) |
| Slight | 8 (16.667) | 13 (28.261) | 15 (32.609) | 36 (25.714) |
| Moderate | 14 (29.167) | 7 (15.217) | 9 (19.565) | 30 (21.429) |
| Severe | 1 (2.083) | 2 (4.348) | 1 (2.174) | 4 (2.857) |
| Extreme/Unable | 0 (0.000) | 0 (0.000) | 0 (0.000) | 0 (0.000) |
|  |  |  |  |  |
| **Day 5** |  |  |  |  |
| n (%) | 43 (82.692) | 45 (93.750) | 45 (90.000) | 133 (88.667) |
| None | 29 (67.442) | 29 (64.444) | 26 (57.778) | 84 (63.158) |
| Slight | 12 (27.907) | 10 (22.222) | 13 (28.889) | 35 (26.316) |
| Moderate | 2 (4.651) | 4 (8.889) | 6 (13.333) | 12 (9.023) |
| Severe | 0 (0.000) | 2 (4.444) | 0 (0.000) | 2 (1.504) |
| Extreme/Unable | 0 (0.000) | 0 (0.000) | 0 (0.000) | 0 (0.000) |
|  |  |  |  |  |
| **Day 15** |  |  |  |  |
| n (%) | 47 (90.385) | 44 (91.667) | 43 (86.000) | 134 (89.333) |
| None | 29 (61.702) | 35 (79.545) | 32 (74.419) | 96 (71.642) |
| Slight | 14 (29.787) | 8 (18.182) | 7 (16.279) | 29 (21.642) |
| Moderate | 3 (6.383) | 1 (2.273) | 4 (9.302) | 8 (5.970) |
| Severe | 0 (0.000) | 0 (0.000) | 0 (0.000) | 0 (0.000) |
| Extreme/Unable | 1 (2.128) | 0 (0.000) | 0 (0.000) | 1 (0.746) |
|  |  |  |  |  |
| **Day 44** |  |  |  |  |
| n (%) | 43 (82.692) | 43 (89.583) | 44 (88.000) | 130 (86.667) |
| None | 30 (69.767) | 28 (65.116) | 30 (68.182) | 88 (67.692) |
| Slight | 9 (20.930) | 9 (20.930) | 12 (27.273) | 30 (23.077) |
| Moderate | 3 (6.977) | 4 (9.302) | 2 (4.545) | 9 (6.923) |
| Severe | 0 (0.000) | 2 (4.651) | 0 (0.000) | 2 (1.538) |
| Extreme/Unable | 1 (2.326) | 0 (0.000) | 0 (0.000) | 1 (0.769) |
|  |  |  |  |  |
| **Week 12** |  |  |  |  |
| n (%) | 43 (82.692) | 41 (85.417) | 41 (82.000) | 125 (83.333) |
| None | 30 (69.767) | 26 (63.415) | 27 (65.854) | 83 (66.400) |
| Slight | 5 (11.628) | 8 (19.512) | 9 (21.951) | 22 (17.600) |
| Moderate | 7 (16.279) | 5 (12.195) | 4 (9.756) | 16 (12.800) |
| Severe | 0 (0.000) | 1 (2.439) | 1 (2.439) | 2 (1.600) |
| Extreme/Unable | 1 (2.326) | 1 (2.439) | 0 (0.000) | 2 (1.600) |
|  |  |  |  |  |
| **Week 24** |  |  |  |  |
| n (%) | 39 (75.000) | 43 (89.583) | 42 (84.000) | 124 (82.667) |
| None | 27 (69.231) | 33 (76.744) | 29 (69.048) | 89 (71.774) |
| Slight | 6 (15.385) | 5 (11.628) | 11 (26.190) | 22 (17.742) |
| Moderate | 5 (12.821) | 4 (9.302) | 1 (2.381) | 10 (8.065) |
| Severe | 0 (0.000) | 1 (2.326) | 1 (2.381) | 2 (1.613) |
| Extreme/Unable | 1 (2.564) | 0 (0.000) | 0 (0.000) | 1 (0.806) |

Abbreviation: NMV/r, nirmatrelvir-ritonavir.

### Supplementary Table 15. EQ-5D-5L Usual Activity scores – overall sample (N=150)

|  | 5-day NMV/r (N=52) | 10-day NMV/r (N=48) | 15-day NMV/r (N=50) | All arms (N=150) |
| --- | --- | --- | --- | --- |
|  | n (%) | n (%) | n (%) | n (%) |
| **Baseline (Day 1)** |  |  |  |  |
| n (%) | 48 (92.308) | 46 (95.833) | 46 (92.000) | 140 (93.333) |
| None | 14 (29.167) | 12 (26.087) | 12 (26.087) | 38 (27.143) |
| Slight | 13 (27.083) | 16 (34.783) | 17 (36.957) | 46 (32.857) |
| Moderate | 13 (27.083) | 11 (23.913) | 12 (26.087) | 36 (25.714) |
| Severe | 3 (6.250) | 4 (8.696) | 2 (4.348) | 9 (6.429) |
| Extreme/Unable | 5 (10.417) | 3 (6.522) | 3 (6.522) | 11 (7.857) |
|  |  |  |  |  |
| **Day 5** |  |  |  |  |
| n (%) | 43 (82.692) | 45 (93.750) | 45 (90.000) | 133 (88.667) |
| None | 18 (41.860) | 16 (35.556) | 20 (44.444) | 54 (40.602) |
| Slight | 16 (37.209) | 21 (46.667) | 16 (35.556) | 53 (39.850) |
| Moderate | 8 (18.605) | 4 (8.889) | 6 (13.333) | 18 (13.534) |
| Severe | 1 (2.326) | 2 (4.444) | 2 (4.444) | 5 (3.759) |
| Extreme/Unable | 0 (0.000) | 2 (4.444) | 1 (2.222) | 3 (2.256) |
|  |  |  |  |  |
| **Day 15** |  |  |  |  |
| n (%) | 47 (90.385) | 44 (91.667) | 43 (86.000) | 134 (89.333) |
| None | 28 (59.574) | 33 (75.000) | 26 (60.465) | 87 (64.925) |
| Slight | 13 (27.660) | 11 (25.000) | 10 (23.256) | 34 (25.373) |
| Moderate | 4 (8.511) | 0 (0.000) | 6 (13.953) | 10 (7.463) |
| Severe | 2 (4.255) | 0 (0.000) | 1 (2.326) | 3 (2.239) |
| Extreme/Unable | 0 (0.000) | 0 (0.000) | 0 (0.000) | 0 (0.000) |
|  |  |  |  |  |
| **Day 44** |  |  |  |  |
| n (%) | 43 (82.692) | 43 (89.583) | 44 (88.000) | 130 (86.667) |
| None | 27 (62.791) | 28 (65.116) | 26 (59.091) | 81 (62.308) |
| Slight | 10 (23.256) | 9 (20.930) | 16 (36.364) | 35 (26.923) |
| Moderate | 5 (11.628) | 5 (11.628) | 2 (4.545) | 12 (9.231) |
| Severe | 1 (2.326) | 1 (2.326) | 0 (0.000) | 2 (1.538) |
| Extreme/Unable | 0 (0.000) | 0 (0.000) | 0 (0.000) | 0 (0.000) |
|  |  |  |  |  |
| **Week 12** |  |  |  |  |
| n (%) | 43 (82.692) | 41 (85.417) | 41 (82.000) | 125 (83.333) |
| None | 31 (72.093) | 27 (65.854) | 28 (68.293) | 86 (68.800) |
| Slight | 6 (13.953) | 12 (29.268) | 11 (26.829) | 29 (23.200) |
| Moderate | 6 (13.953) | 1 (2.439) | 1 (2.439) | 8 (6.400) |
| Severe | 0 (0.000) | 1 (2.439) | 1 (2.439) | 2 (1.600) |
| Extreme/Unable | 0 (0.000) | 0 (0.000) | 0 (0.000) | 0 (0.000) |
|  |  |  |  |  |
| **Week 24** |  |  |  |  |
| n (%) | 39 (75.000) | 43 (89.583) | 42 (84.000) | 124 (82.667) |
| None | 27 (69.231) | 30 (69.767) | 28 (66.667) | 85 (68.548) |
| Slight | 5 (12.821) | 9 (20.930) | 8 (19.048) | 22 (17.742) |
| Moderate | 6 (15.385) | 3 (6.977) | 5 (11.905) | 14 (11.290) |
| Severe | 0 (0.000) | 1 (2.326) | 1 (2.381) | 2 (1.613) |
| Extreme/Unable | 1 (2.564) | 0 (0.000) | 0 (0.000) | 1 (0.806) |

Abbreviation: NMV/r, nirmatrelvir-ritonavir.

### Supplementary Table 16. EQ-5D-5L Pain/Discomfort scores – overall sample (N=150)

|  | 5-day NMV/r (N=52) | 10-day NMV/r (N=48) | 15-day NMV/r (N=50) | All arms (N=150) |
| --- | --- | --- | --- | --- |
|  | n (%) | n (%) | n (%) | n (%) |
| **Baseline (Day 1)** |  |  |  |  |
| n (%) | 48 (92.308) | 46 (95.833) | 46 (92.000) | 140 (93.333) |
| None | 5 (10.417) | 3 (6.522) | 6 (13.043) | 14 (10.000) |
| Slight | 20 (41.667) | 22 (47.826) | 17 (36.957) | 59 (42.143) |
| Moderate | 19 (39.583) | 17 (36.957) | 19 (41.304) | 55 (39.286) |
| Severe | 4 (8.333) | 4 (8.696) | 4 (8.696) | 12 (8.571) |
| Extreme/Unable | 0 (0.000) | 0 (0.000) | 0 (0.000) | 0 (0.000) |
|  |  |  |  |  |
| **Day 5** |  |  |  |  |
| n (%) | 43 (82.692) | 45 (93.750) | 45 (90.000) | 133 (88.667) |
| None | 16 (37.209) | 13 (28.889) | 14 (31.111) | 43 (32.331) |
| Slight | 18 (41.860) | 26 (57.778) | 21 (46.667) | 65 (48.872) |
| Moderate | 8 (18.605) | 4 (8.889) | 9 (20.000) | 21 (15.789) |
| Severe | 1 (2.326) | 2 (4.444) | 1 (2.222) | 4 (3.008) |
| Extreme/Unable | 0 (0.000) | 0 (0.000) | 0 (0.000) | 0 (0.000) |
|  |  |  |  |  |
| **Day 15** |  |  |  |  |
| n (%) | 47 (90.385) | 44 (91.667) | 43 (86.000) | 134 (89.333) |
| None | 26 (55.319) | 29 (65.909) | 24 (55.814) | 79 (58.955) |
| Slight | 20 (42.553) | 14 (31.818) | 14 (32.558) | 48 (35.821) |
| Moderate | 1 (2.128) | 1 (2.273) | 4 (9.302) | 6 (4.478) |
| Severe | 0 (0.000) | 0 (0.000) | 1 (2.326) | 1 (0.746) |
| Extreme/Unable | 0 (0.000) | 0 (0.000) | 0 (0.000) | 0 (0.000) |
|  |  |  |  |  |
| **Day 44** |  |  |  |  |
| n (%) | 43 (82.692) | 43 (89.583) | 44 (88.000) | 130 (86.667) |
| None | 27 (62.791) | 22 (51.163) | 20 (45.455) | 69 (53.077) |
| Slight | 13 (30.233) | 14 (32.558) | 16 (36.364) | 43 (33.077) |
| Moderate | 3 (6.977) | 6 (13.953) | 7 (15.909) | 16 (12.308) |
| Severe | 0 (0.000) | 1 (2.326) | 1 (2.273) | 2 (1.538) |
| Extreme/Unable | 0 (0.000) | 0 (0.000) | 0 (0.000) | 0 (0.000) |
|  |  |  |  |  |
| **Week 12** |  |  |  |  |
| n (%) | 43 (82.692) | 41 (85.417) | 41 (82.000) | 125 (83.333) |
| None | 28 (65.116) | 23 (56.098) | 20 (48.780) | 71 (56.800) |
| Slight | 6 (13.953) | 12 (29.268) | 14 (34.146) | 32 (25.600) |
| Moderate | 9 (20.930) | 4 (9.756) | 3 (7.317) | 16 (12.800) |
| Severe | 0 (0.000) | 2 (4.878) | 4 (9.756) | 6 (4.800) |
| Extreme/Unable | 0 (0.000) | 0 (0.000) | 0 (0.000) | 0 (0.000) |
|  |  |  |  |  |
| **Week 24** |  |  |  |  |
| n (%) | 39 (75.000) | 43 (89.583) | 42 (84.000) | 124 (82.667) |
| None | 26 (66.667) | 25 (58.140) | 27 (64.286) | 78 (62.903) |
| Slight | 7 (17.949) | 12 (27.907) | 6 (14.286) | 25 (20.161) |
| Moderate | 5 (12.821) | 5 (11.628) | 8 (19.048) | 18 (14.516) |
| Severe | 1 (2.564) | 0 (0.000) | 1 (2.381) | 2 (1.613) |
| Extreme/Unable | 0 (0.000) | 1 (2.326) | 0 (0.000) | 1 (0.806) |

Abbreviation: NMV/r, nirmatrelvir-ritonavir.

### Supplementary Table 17. EQ-5D-5L Anxiety/Depression scores – overall sample (N=150)

| Visit | 5-day NMV/r (N=52) | 10-day NMV/r (N=48) | 15-day NMV/r (N=50) | All arms (N=150) |
| --- | --- | --- | --- | --- |
|  | n (%) | n (%) | n (%) | n (%) |
| **Baseline (Day 1)** |  |  |  |  |
| n (%) | 48 (92.308) | 46 (95.833) | 46 (92.000) | 140 (93.333) |
| None | 33 (68.750) | 22 (47.826) | 18 (39.130) | 73 (52.143) |
| Slight | 10 (20.833) | 17 (36.957) | 15 (32.609) | 42 (30.000) |
| Moderate | 5 (10.417) | 5 (10.870) | 7 (15.217) | 17 (12.143) |
| Severe | 0 | 2 (4.348) | 6 (13.043) | 8 (5.714) |
| Extreme/Unable | 0 | 0 | 0 | 0 |
|  |  |  |  |  |
| **Day 5** |  |  |  |  |
| n (%) | 43 (82.692) | 45 (93.750) | 45 (90.000) | 133 (88.667) |
| None | 32 (74.419) | 29 (64.444) | 26 (57.778) | 87 (65.414) |
| Slight | 9 (20.930) | 11 (24.444) | 14 (31.111) | 34 (25.564) |
| Moderate | 1 (2.326) | 2 (4.444) | 5 (11.111) | 8 (6.015) |
| Severe | 1 (2.326) | 2 (4.444) | 0 | 3 (2.256) |
| Extreme/Unable | 0 | 1 (2.222) | 0 | 1 (0.752) |
|  |  |  |  |  |
| **Day 15** |  |  |  |  |
| n (%) | 47 (90.385) | 44 (91.667) | 43 (86.000) | 134 (89.333) |
| None | 37 (78.723) | 34 (77.273) | 31 (72.093) | 102 (76.119) |
| Slight | 8 (17.021) | 8 (18.182) | 8 (18.605) | 24 (17.910) |
| Moderate | 2 (4.255) | 1 (2.273) | 4 (9.302) | 7 (5.224) |
| Severe | 0 | 0 | 0 | 0 |
| Extreme/Unable | 0 | 1 (2.273) | 0 | 1 (0.746) |
|  |  |  |  |  |
| **Day 44** |  |  |  |  |
| n (%) | 43 (82.692) | 43 (89.583) | 44 (88.000) | 130 (86.667) |
| None | 36 (83.721) | 30 (69.767) | 27 (61.364) | 93 (71.538) |
| Slight | 5 (11.628) | 9 (20.930) | 13 (29.545) | 27 (20.769) |
| Moderate | 2 (4.651) | 2 (4.651) | 3 (6.818) | 7 (5.385) |
| Severe | 0 | 2 (4.651) | 0 | 2 (1.538) |
| Extreme/Unable | 0 | 0 | 1 (2.273) | 1 (0.769) |
|  |  |  |  |  |
| **Week 12** |  |  |  |  |
| n (%) | 43 (82.692) | 41 (85.417) | 41 (82.000) | 125 (83.333) |
| None | 32 (74.419) | 32 (78.049) | 31 (75.610) | 95 (76.000) |
| Slight | 9 (20.930) | 5 (12.195) | 6 (14.634) | 20 (16.000) |
| Moderate | 2 (4.651) | 2 (4.878) | 2 (4.878) | 6 (4.800) |
| Severe | 0 | 1 (2.439) | 2 (4.878) | 3 (2.400) |
| Extreme/Unable | 0 | 1 (2.439) | 0 | 1 (0.800) |
|  |  |  |  |  |
| **Week 24** |  |  |  |  |
| n (%) | 39 (75.000) | 43 (89.583) | 42 (84.000) | 124 (82.667) |
| None | 31 (79.487) | 33 (76.744) | 34 (80.952) | 98 (79.032) |
| Slight | 5 (12.821) | 7 (16.279) | 3 (7.143) | 15 (12.097) |
| Moderate | 2 (5.128) | 1 (2.326) | 4 (9.524) | 7 (5.645) |
| Severe | 1 (2.564) | 1 (2.326) | 1 (2.381) | 3 (2.419) |
| Extreme/Unable | 0 | 1 (2.326) | 0 | 1 (0.806) |

Abbreviation: NMV/r, nirmatrelvir-ritonavir.

### Supplementary Table 18. EQ-5D-5L Index scores

| Visit Date | 5-day NMV/r (N=52) | 10-day NMV/r (N=48) | 15-day NMV/r (N=50) | All arms (N=150) |
| --- | --- | --- | --- | --- |
| **Baseline (Day 1)** |  |  |  |  |
| *Observed* |  |  |  |  |
| n | 48 | 46 | 46 | 140 |
| Mean (SD) | 0.667 (0.199) | 0.650 (0.202) | 0.634 (0.225) | 0.650 (0.207) |
| SE | 0.029 | 0.030 | 0.033 | 0.018 |
| Median (range) | 0.726 (0.100, 1.000) | 0.735 (0.054, 1.000) | 0.707 (-0.015, 1.000) | 0.717 (-0.015, 1.000) |
| 95% CI | [0.609, 0.724] | [0.590, 0.709] | [0.568, 0.701] | [0.616, 0.685] |
|  |  |  |  |  |
| **Day 5** |  |  |  |  |
| *Observed* |  |  |  |  |
| n | 43 | 45 | 45 | 133 |
| Mean (SD) | 0.794 (0.151) | 0.737 (0.203) | 0.761 (0.181) | 0.763 (0.180) |
| SE | 0.023 | 0.030 | 0.027 | 0.016 |
| Median (range) | 0.767 (0.441, 1.000) | 0.795 (0.036, 1.000) | 0.795 (0.219, 1.000) | 0.778 (0.036, 1.000) |
| 95% CI | [0.747, 0.840] | [0.676, 0.798] | [0.707, 0.815] | [0.732, 0.794] |
| *Change from baseline* |  |  |  |  |
| n | 42 | 43 | 41 | 126 |
| Mean (SD) | 0.110 (0.190) | 0.091 (0.135) | 0.117 (0.195) | 0.106 (0.174) |
| SE | 0.029 | 0.021 | 0.030 | 0.016 |
| Median (range) | 0.057 (-0.277, 0.513) | 0.059 (-0.212, 0.537) | 0.083 (-0.297, 0.694) | 0.072 (-0.297, 0.694) |
| 95% CI | [0.051, 0.169] | [0.049, 0.132] | [0.055, 0.178] | [0.075, 0.136] |
|  |  |  |  |  |
| **Day 15** |  |  |  |  |
| *Observed* |  |  |  |  |
| n | 47 | 44 | 43 | 134 |
| Mean (SD) | 0.834 (0.173) | 0.879 (0.164) | 0.850 (0.175) | 0.854 (0.171) |
| SE | 0.025 | 0.025 | 0.027 | 0.015 |
| Median (range) | 0.809 (0.238, 1.000) | 0.879 (0.131, 1.000) | 0.848 (0.246, 1.000) | 0.877 (0.131, 1.000) |
| 95% CI | [0.783, 0.885] | [0.829, 0.929] | [0.796, 0.904] | [0.825, 0.883] |
| *Change from baseline* |  |  |  |  |
| n | 45 | 43 | 40 | 128 |
| Mean (SD) | 0.177 (0.206) | 0.224 (0.193) | 0.221 (0.189) | 0.206 (0.196) |
| SE | 0.031 | 0.029 | 0.030 | 0.017 |
| Median (range) | 0.163 (-0.205, 0.806) | 0.204 (-0.197, 0.742) | 0.205 (-0.117, 0.767) | 0.204 (-0.205, 0.806) |
| 95% CI | [0.115, 0.239] | [0.164, 0.283] | [0.160, 0.281] | [0.172, 0.241] |
|  |  |  |  |  |
| **Day 44** |  |  |  |  |
| *Observed* |  |  |  |  |
| n | 43 | 43 | 44 | 130 |
| Mean (SD) | 0.861 (0.166) | 0.814 (0.200) | 0.806 (0.164) | 0.827 (0.178) |
| SE | 0.025 | 0.030 | 0.025 | 0.016 |
| Median (range) | 1.000 (0.336, 1.000) | 0.837 (0.276, 1.000) | 0.837 (0.330, 1.000) | 0.837 (0.276, 1.000) |
| 95% CI | [0.810, 0.912] | [0.752, 0.875] | [0.756, 0.856] | [0.796, 0.858] |
| *Change from baseline* |  |  |  |  |
| n | 41 | 42 | 41 | 124 |
| Mean (SD) | 0.184 (0.191) | 0.153 (0.221) | 0.169 (0.192) | 0.169 (0.201) |
| SE | 0.030 | 0.034 | 0.030 | 0.018 |
| Median (range) | 0.191 (-0.221, 0.668) | 0.160 (-0.319, 0.742) | 0.163 (-0.204, 0.669) | 0.163 (-0.319, 0.742) |
| 95% CI | [0.124, 0.245] | [0.084, 0.222] | [0.108, 0.230] | [0.133, 0.204] |
|  |  |  |  |  |
| **Week 12** |  |  |  |  |
| *Observed* |  |  |  |  |
| n | 43 | 41 | 41 | 125 |
| Mean (SD) | 0.856 (0.180) | 0.809 (0.230) | 0.806 (0.210) | 0.824 (0.207) |
| SE | 0.027 | 0.036 | 0.033 | 0.019 |
| Median (range) | 1.000 (0.336, 1.000) | 0.837 (0.221, 1.000) | 0.837 (0.277, 1.000) | 0.879 (0.221, 1.000) |
| 95% CI | [0.800, 0.911] | [0.736, 0.881] | [0.740, 0.872] | [0.787, 0.861] |
| *Change from baseline* |  |  |  |  |
| n | 41 | 40 | 39 | 120 |
| Mean (SD) | 0.198 (0.221) | 0.168 (0.274) | 0.166 (0.211) | 0.177 (0.236) |
| SE | 0.035 | 0.043 | 0.034 | 0.021 |
| Median (range) | 0.204 (-0.161, 0.668) | 0.163 (-0.437, 0.946) | 0.204 (-0.321, 0.669) | 0.168 (-0.437, 0.946) |
| 95% CI | [0.128, 0.268] | [0.080, 0.255] | [0.097, 0.234] | [0.135, 0.220] |
|  |  |  |  |  |
| **Week 24** |  |  |  |  |
| *Observed* |  |  |  |  |
| n | 39 | 43 | 42 | 124 |
| Mean (SD) | 0.832 (0.199) | 0.844 (0.242) | 0.860 (0.182) | 0.846 (0.209) |
| SE | 0.032 | 0.037 | 0.028 | 0.019 |
| Median (range) | 0.877 (0.280, 1.000) | 1.000 (-0.116, 1.000) | 1.000 (0.206, 1.000) | 1.000 (-0.116, 1.000) |
| 95% CI | [0.768, 0.897] | [0.770, 0.919] | [0.803, 0.917] | [0.809, 0.883] |
| *Change from baseline* |  |  |  |  |
| n | 37 | 42 | 39 | 118 |
| Mean (SD) | 0.178 (0.228) | 0.213 (0.237) | 0.221 (0.207) | 0.204 (0.223) |
| SE | 0.037 | 0.037 | 0.033 | 0.021 |
| Median (range) | 0.163 (-0.346, 0.682) | 0.188 (-0.379, 0.946) | 0.214 (-0.265, 0.704) | 0.204 (-0.379, 0.946) |
| 95% CI | [0.102, 0.254] | [0.139, 0.286] | [0.154, 0.288] | [0.164, 0.245] |

Abbreviations: CI, confidence interval; NMV/r, nirmatrelvir-ritonavir; SD, standard deviation; SE, standard error.

## Supplementary figures

### Supplementary Figure 1. SF-36 domain scores by treatment arm

Scores for each SF-36 domain in the overall sample (N=150) at each study visit for participants in the 5-day NMV/r, 10-day NMV/r, and 15-day NMV/r treatment arms.

Abbreviations: NMV/r, nirmatrelvir-ritonavir; SD, standard deviation; SF-36, 36-Item Short Form Health Survey.

### Supplementary Figure 2. SF-36 domain scores by IC severity and treatment arm

Scores for each SF-36 domain by severe IC and non-severe IC subpopulation and treatment arm at each study visit.

Abbreviations: BL, baseline; D, day; IC, immunocompromise; NMV/r, nirmatrelvir-ritonavir; SD, standard deviation; SF-36, 36-Item Short Form Health Survey; W, week.

### Supplementary Figure 3. SF-36 Physical Component Summary scores by treatment arm in the overall sample

SF-36 Physical Component Summary scores by study day in EPIC-IC participants treated with 5-day, 10-day, or 15-day NMV/r, and population norms. Scores are shown for the overall evaluable sample (N=150). Dotted line depicts an age-matched US general population norm, calculated as the unweighted average of male and female mean scores for participants ages 55–64 years in the National Health Measurement Study [7].

Abbreviations: PCS, Physical Component Summary; NMV/r, nirmatrelvir-ritonavir; SD, standard deviation; SF-36, 36-Item Short Form Survey version 2 - acute form.

### Supplementary Figure 4. SF-36 Physical Component Summary scores by treatment arm in severe IC and non-severe IC subpopulations

SF-36 Physical Component Summary scores by study day in EPIC-IC participants treated with 5-day, 10-day, or 15-day NMV/r, and population norms. Scores are shown for subpopulations with severe IC (n=57) and non-severe IC (n=93). Dotted lines depict an age-matched US general population norm, calculated as the unweighted average of male and female mean scores for participants ages 55–64 years in the National Health Measurement Study [7].

Abbreviations: D, day; IC, immunocompromise; PCS, Physical Component Summary; NMV/r, nirmatrelvir-ritonavir; SD, standard deviation; SF-36, 36-Item Short Form Survey version 2 - acute form; W, week.

### Supplementary Figure 5. SF-36 Mental Component Summary scores by treatment arm in the overall sample

SF-36 Mental Component Summary scores by study day in EPIC-IC participants treated with 5-day, 10-day, or 15-day NMV/r, and population norms. Scores are shown for the overall sample. Dotted line depicts an age-matched US general population norm, calculated as the unweighted average of male and female mean scores for participants ages 55–64 years in the National Health Measurement Study [7].

Abbreviations: MCS, Mental Component Summary; NMV/r, nirmatrelvir-ritonavir; SD, standard deviation; SF-36, 36-Item Short Form Survey version 2 - acute form.

### Supplementary Figure 6. SF-36 Mental Component Summary scores by treatment arm in severe IC and non-severe IC subpopulations

SF-36 Mental Component Summary scores by study day in EPIC-IC participants treated with 5-day, 10-day, or 15-day NMV/r, and population norms. Scores are shown for subpopulations with severe IC (n=57) and non-severe IC (n=93). Dotted lines depict an age-matched US general population norm, calculated as the unweighted average of male and female mean scores for participants ages 55–64 years in the National Health Measurement Study [7].

Abbreviations: D, day; IC, immunocompromise; PCS, Physical Component Summary; NMV/r, nirmatrelvir-ritonavir; SD, standard deviation; SF-36, 36-Item Short Form Survey version 2 - acute form; W, week.

### Supplementary Figure 7. Participants reporting ‘no problems’/‘none’ in EQ-5D-5L dimensions across treatment arms and by treatment arm

Percentage of participants in the overall sample (A) across treatment arms and (B-F) by treatment arm reporting ‘no problems’/‘none’ in EQ-5D-5L dimensions at each study visit.

Abbreviation: NMV/r, nirmatrelvir-ritonavir.

### Supplementary Figure 8. Participants reporting ‘no problems’/’none’ in EQ-5D-5L domain scores by IC severity and treatment arm

Percentage of participants in the severe IC (filled shapes) and non-severe IC (open shapes) subpopulations reporting ‘no problems’/‘none’ in EQ-5D-5L domains at each study visit.

Abbreviations: EQ-5D-5L, European Quality of Life 5 Dimensions 5 Level Version; IC, immunocompromise; NMV/r, nirmatrelvir-ritonavir.

### Supplementary Figure 9. EQ-5D-5L Index scores by treatment arm in the overall sample

EQ-5D-5L Index scores by study day in EPIC-IC participants treated with 5-day, 10-day, or 15-day NMV/r. The general population norm is the mean index score of a UK population with various health conditions (0.65) [8].

Abbreviations: NMV/r, nirmatrelvir-ritonavir; SD, standard deviation.

### Supplementary Figure 10. EQ-5D-5L Index scores by treatment arm in severe IC and non-severe IC subpopulations

EQ-5D-5L Index scores by study day in EPIC-IC participants treated with 5-day, 10-day, or 15-day NMV/r, shown for subpopulations with severe IC (n=57) and non-severe IC (n=93). The general population norm is the mean index score of a UK population with various health conditions (0.65) [8].

Abbreviations: BL, baseline; D, day; IC, immunocompromise; NMV/r, nirmatrelvir-ritonavir; SD, standard deviation; W, week.

# References

1. US Centers for Disease Control and Prevention. Underlying Conditions and the Higher Risk for Severe COVID-19. 2025. Updated 06-Feb-2025; Accessed 12-Jun-2025. Available from: <https://www.cdc.gov/covid/hcp/clinical-care/underlying-conditions.html>

2. Weinstein E, Paredes R, Gardner A, Almas M, Baniecki ML, Guan S, et al. Extended nirmatrelvir–ritonavir treatment durations for immunocompromised patients with COVID-19 (EPIC-IC): a placebo-controlled, randomised, double-blind, phase 2 trial. Lancet Infect Dis. 2025. <https://doi.org/10.1016/S1473-3099(25)00221-X>

3. Ware JE, Jr. , Kosinski M, J.B. B, Turner-Bowker DM, Gandek B, Maruish ME. User’s Manual for the SF-36v2® Health Survey. 2nd ed. In: QualityMetric Incorporated; Lincoln, RI; 2007.

4. Herdman M, Gudex C, Lloyd A, Janssen MF, Kind P, Parkin D, et al. Development and preliminary testing of the new five-level version of EQ-5D (EQ-5D-5L). Qual Life Res. 2011;20(10):1727-36. <https://doi.org/10.1007/s11136-011-9903-x>

5. van Hout B, Janssen MF, Feng Y-S, Kohlmann T, Busschbach J, Golicki D, et al. Interim Scoring for the EQ-5D-5L: Mapping the EQ-5D-5L to EQ-5D-3L Value Sets. Value in Health. 2012;15(5):708-15. <https://doi.org/https://doi.org/10.1016/j.jval.2012.02.008>

6. Fitzmaurice GM, Laird NM, Ware JH. Applied Longitudinal Analysis. 2nd ed. In: John Wiley & Sons, Inc.; 2011.

7. Maglinte GA, Hays RD, Kaplan RM. US general population norms for telephone administration of the SF-36v2. J Clin Epidemiol. 2012;65(5):497-502. <https://doi.org/10.1016/j.jclinepi.2011.09.008>

8. Mulhern B, Feng Y, Shah K, Janssen MF, Herdman M, van Hout B, Devlin N. Comparing the UK EQ-5D-3L and English EQ-5D-5L Value Sets. Pharmacoeconomics. 2018;36(6):699-713. <https://doi.org/10.1007/s40273-018-0628-3>
